# A ruthenium single atom nanozyme-based antibiotic for the treatment of otitis media caused by *Staphylococcus aureus*

**DOI:** 10.3389/fchem.2024.1439039

**Published:** 2024-08-28

**Authors:** Jie Wang, Rui Gong, Ming Yang, Xi Wu, Ziwei Li, Haibing Huang, Xiyun Yan, Daji Wang

**Affiliations:** ^1^ Nanozyme Synthesis Center, Key Laboratory of Quantitative Synthetic Biology, Shenzhen Institute of Synthetic Biology, Shenzhen Institutes of Advanced Technology, Chinese Academy of Sciences, Shenzhen, China; ^2^ Faculty of Synthetic Biology, Shenzhen Institute of Advanced Technology, Chinese Academy of Sciences, Shenzhen, China; ^3^ Department of Otolaryngology, Shenzhen People’s Hospital (The Second Clinical Medical College, Jinan University, The First Affiliated Hospital, Southern University of Science and Technology), Shenzhen, China; ^4^ Department of Clinical Laboratory, Shenshan Central Hospital, Sun Yat-Sen Memorial Hospital, Sun Yat-Sen University, Shanwei, China; ^5^ CAS Engineering Laboratory for Nanozyme, Key Laboratory of Biomacromolecules, Institute of Biophysics, Chinese Academy of Sciences, Beijing, China; ^6^ Nanozyme Laboratory in Zhongyuan, Henan Academy of Innovations in Medical Science, Zhengzhou, China

**Keywords:** single atom nanozyme, ruthenium, peroxidase, *Staphylococcus aureus*, otitis media, nanoantibiotics

## Abstract

*Staphylococcus aureus* (*S. aureus*) infection is a primary cause of otitis media (OM), the most common disease for which children are prescribed antibiotics. However, the abuse of antibiotics has led to a global increase in antimicrobial resistance (AMR). Nanozymes, as promising alternatives to traditional antibiotics, are being extensively utilized to combat AMR. Here, we synthesize a series of single-atom nanozymes (metal-C_3_N_4_ SANzymes) by loading four metals (Ag, Fe, Cu, Ru) with antibacterial properties onto a crystalline g-C_3_N_4_. These metal-C_3_N_4_ display a rob-like morphology and well-dispersed metal atoms. Among them, Ru-C_3_N_4_ demonstrates the optimal peroxidase-like activity (285.3 U mg^–1^), comparable to that of horseradish peroxidase (267.7 U mg^–1^). *In vitro* antibacterial assays reveal that Ru-C_3_N_4_ significantly inhibits *S. aureus* growth compared with other metal-C_3_N_4_ even at a low concentration (0.06 mg mL^–1^). Notably, Ru-C_3_N_4_ acts as a narrow-spectrum nanoantibiotic with relative specificity against Gram-positive bacteria. Biofilms formed by *S. aureus* are easily degraded by Ru-C_3_N_4_ due to its high peroxidase-like activity. *In vivo*, Ru-C_3_N_4_ effectively eliminates *S. aureus* and relieves ear inflammation in OM mouse models. However, untreated OM mice eventually develop hearing impairment. Due to its low metal load, Ru-C_3_N_4_ does not exhibit significant toxicity to blood, liver, or kidney. In conclusion, this study presents a novel SANzyme-based antibiotic that can effectively eliminate *S. aureus* and treat *S. aureus*-induced OM.

## 1 Introduction

Otitis media (OM) is a prevalent disease that is frequently diagnosed and treated by pediatricians. It is estimated that nearly half of all children will experience at least one episode of ear infection by the time they reach their second birthday (Paul and Moreno, 2020). The global incidence rate of OM is approximately 16% (Elzinga et al., 2021), underscoring the significant impact of OM on pediatric health and the urgent need for effective treatment strategies. OM can be manifested in the form of acute OM (AOM), OM with effusion, persistent as recurrent AOM (rAOM) and chronic suppurative otitis media (CSOM) (Hoberman et al., 2021). If left untreated, AOM will progress to CSOM, leading to serious complications such as mastoiditis, labyrinthitis, facial nerve palsy, meningitis, and cerebral abscesses (Holm et al., 2020). These complications are particularly problematic in low-income countries (Peñaranda et al., 2023), where an estimated 21,000 people die annually from OM complications (Monasta et al., 2012). The estimated global prevalence of OM patients with hearing loss is approximately 30 (ranging from 0.7 to 95) per 10,000 individuals (Li et al., 2022).

The management of uncomplicated OM typically involves the cleansing of external auditory canal and antibiotics application. Topical aminoglycosides and quinolones are commonly used as first-line antibiotics (Nawaz et al., 2023). However, the overprescription of these drugs significantly contributes to the development of AMR (Dadgostar, 2019; Huemer et al., 2020). It is estimated that 300 million individuals will experience premature mortality due to AMR within the next 35 years (Ahmed et al., 2024). The Gram-positive bacterium *Staphylococcus aureus* (*S. aureus*) infection is recognized as a primary cause of OM(Mohammed and Abdullah, 2020), constituting 13% (171 out of 1309) of total isolates (Roland et al., 2005). During infection, planktonic *S. aureus* invades the middle ear via the eustachian tube, leading to the inflammation of the middle ear mucosa and tympanic cavity. Independent *S. aureus* can form biofilms within the middle ear and attach to the surface of middle ear mucosal cells (Abu Bakar et al., 2018). In this biofilm state, the resistance of *S. aureus* to antibiotics significantly increases. Antibiotics tend to accumulate within the biofilm matrix, a barrier that inhibits their penetration into the deeper bacterial layers. Consequently, this restriction hampers antibiotics mobility within the matrix, thereby compromising their delivery to the inner layers of the biofilms (Coppens et al., 2023). For instance, vancomycin is the first-line drug for treating infections associated with *S. aureus*. In recent years, there has been a notable reduction in the sensitivity of *S. aureus* to vancomycin due to its increased usage and dosage (Cong et al., 2020). Notably, the resistant phenotype of *S. aureus* can rapidly switch to a susceptible phenotype when the biofilms are disrupted (Parastan et al., 2020). Destructive enzymes have been proposed as an effective agent for eliminating adhesions and preventing bacterial accumulation within the biofilm structure. Protease, hyaluronidase, lysostaphin, and peroxidase can destroy bacteria-formed biofilms through various mechanisms (Rosenthal et al., 2014; Wei et al., 2021; Song et al., 2024b). However, certain intrinsic limitations such as high cost, low catalytic stability, and complex preparation process of natural enzymes restrict their practical application in the antibacterial field (Zhang et al., 2020).

Nanozymes are emerging as promising alternatives to natural enzymes due to their inherent enzymatic properties and activities. They exhibit kinetics and catalytic mechanisms that closely mimic those of natural enzymes. Unlike conventional antibiotics, nanozymes induce the development of AMR at a lower frequency, attributed to their excellent membrane permeability and catalytic activity (Mei et al., 2021; Feng et al., 2022). Furthermore, their enzyme-like properties effectively eliminate bacterial biofilms (Gao et al., 2014; Liang et al., 2020; Xu et al., 2021). Antibacterial nanozymes encompass metal- or nonmetal-based nanoparticles, nanoclusters, and single-atom nanozymes (SANzymes) (Xiong et al., 2019; Meng et al., 2022; Lan et al., 2023). Among these categories, SANzymes display optimal catalytic activity due to their uniformly distributed active sites and maximum atomic utilization (nearly 100%) (Tang et al., 2022). Compared to conventional nanoparticles, the low metal atom loading also improves the biocompatibility of SANzymes (Zhu et al., 2023). Recently, a variety of metal-based nanozymes have been demonstrated to have promising antibacterial efficacy, such as Fe, Ru, Ag, Cu, Pt and Ir (Mei et al., 2021). Compared to other enzyme-based therapeutic approaches, nanozymes can generate a higher intensity of reactive oxygen species (ROS) to easily disrupt biofilms and induce bacterial death. Therefore, the design and synthesis of efficient antibacterial nanozymes hold significant potential for addressing AMR.

**SCHEME 1:**
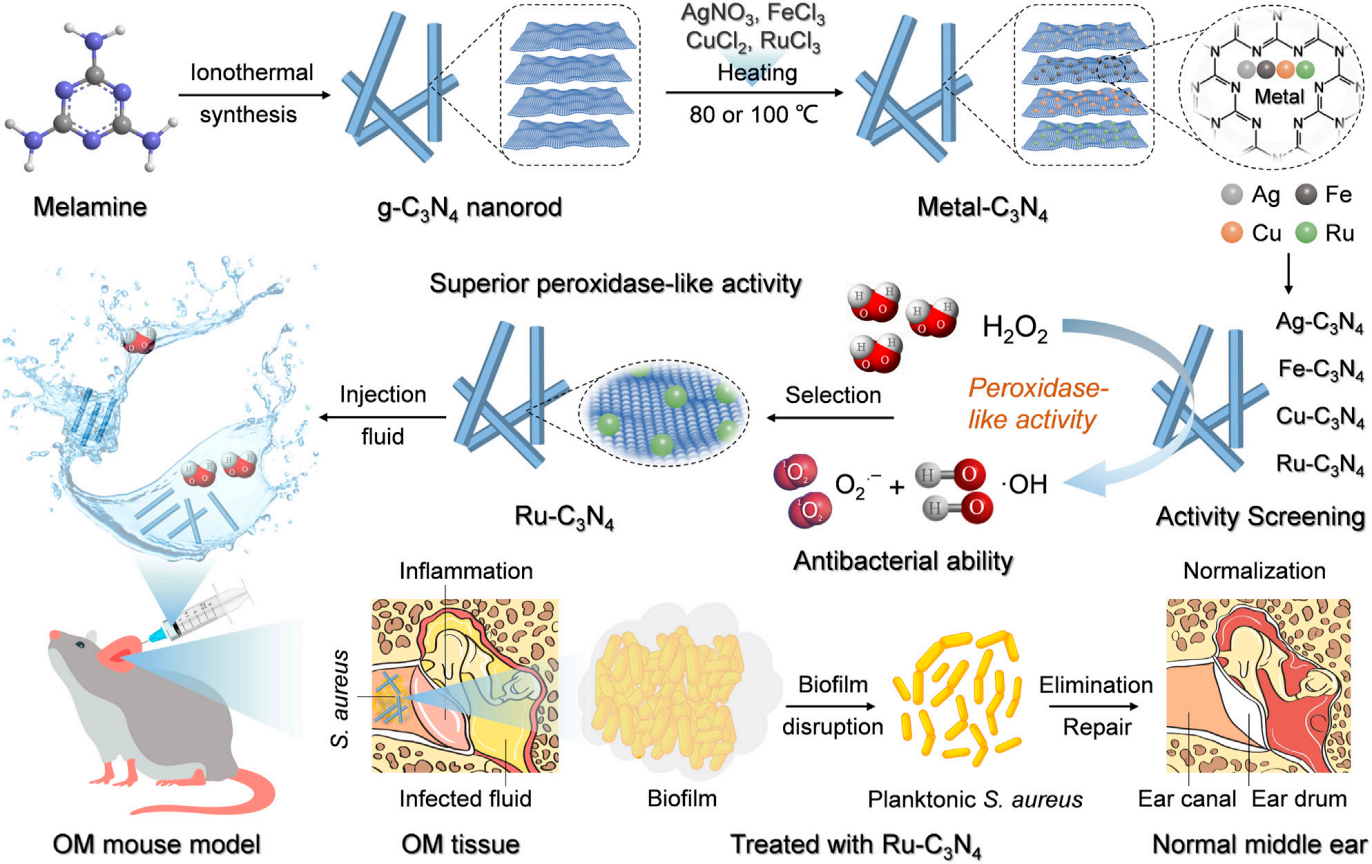
The schematic illustration of metal-C_3_N_4_ preparation and their treatment of *S. aureus*-induced OM model.

In this study, we successfully synthesized four SANzymes with similar geometries and well-defined atomic metals to replace natural peroxidase in the treatment of *S. aureus*-induced OM. We selected Ag, Fe, Cu, and Ru as the metal catalytic sites due to their superior antibacterial properties (Gao et al., 2017; Huang et al., 2017; Xi et al., 2020; Wang et al., 2021). The crystalline g-C_3_N_4_ nanorods were synthesized and utilized as the carrier of single atoms to improve the metal loading and catalytic activity. Meanwhile, its excellent biosafety further expanded its application (Pourmadadi et al., 2023). Characterization data showed that four metal-C_3_N_4_ exhibited a rod-like structure, with monodispersed metal atoms on the C_3_N_4_ carrier. We then conducted a systematic investigation of the peroxidase-like properties of these metal-C_3_N_4_, including catalytic activity, catalytic efficiency, substrate selectivity, and ROS production capacity. Among these SANzymes, Ru-C_3_N_4_ demonstrated superior peroxidase-like activity (285.3 U mg^–1^), comparable to that of HRP (267.7 U mg^–1^). Furthermore, Ru-C_3_N_4_ exhibited optimal efficacy in inhibiting *S. aureus* growth and disrupting biofilms. We finally conducted a comprehensive evaluation of the therapeutic effect of Ru-C_3_N_4_ on OM induced by *S. aureus in vivo* (Scheme 1). In summary, this study provides an effective SANzyme for combating *S. aureus* and its associated OM.

## 2 Results and discussion

### 2.1 Synthesis and characterization of metal-C_3_N_4_

**FIGURE 1:**
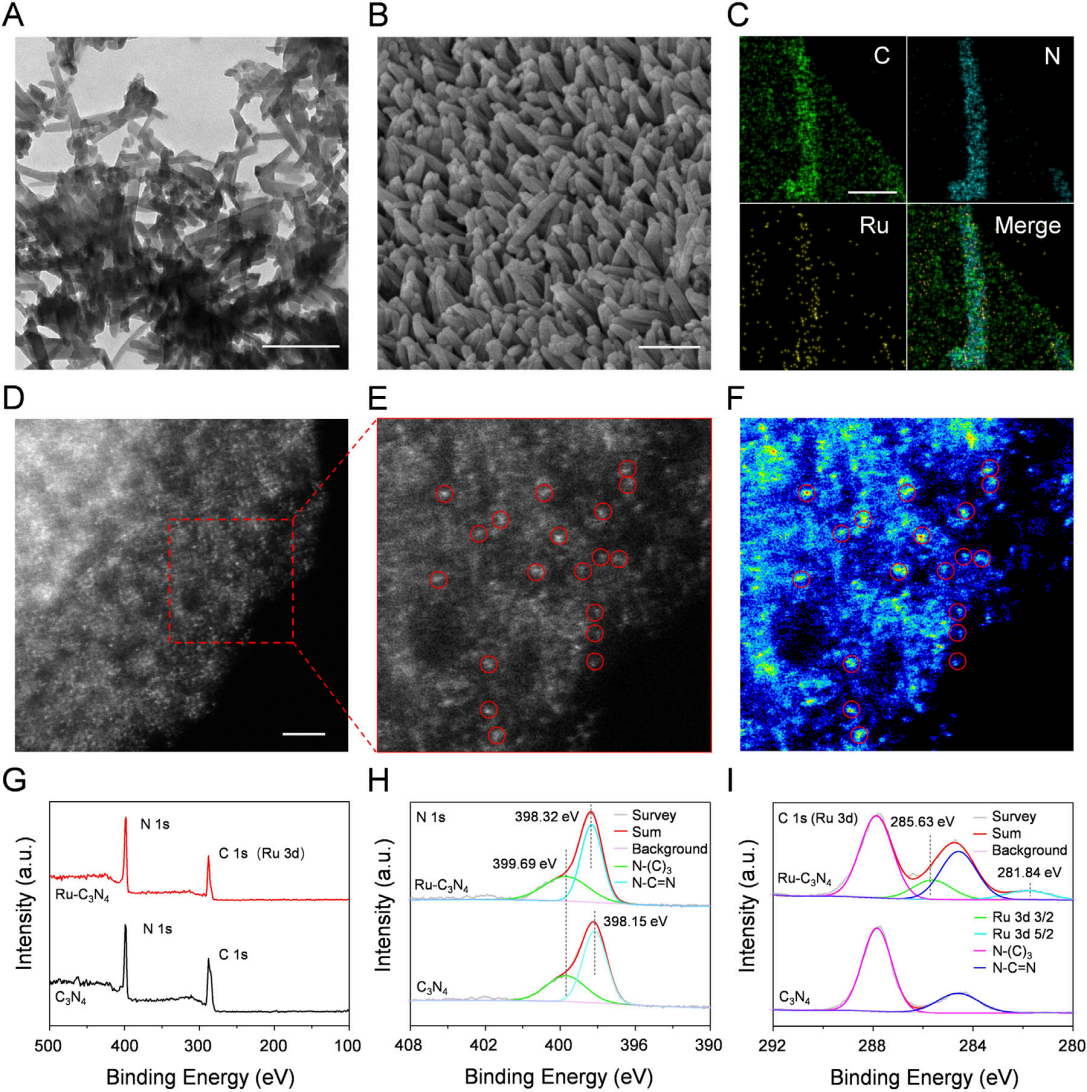
The characterization of Ru-C_3_N_4_. **(A)** TEM image of Ru-C_3_N_4_. Scale bar = 200 nm. **(B)** SEM image of Ru-C_3_N_4_. Scale bar = 200 nm. **(C)** EDS mapping images of Ru-C_3_N_4_. Scale bar = 80 nm. **(D–E)** HAADF-STEM image and the enlarged image of Ru-C_3_N_4_. Scale bar = 2 nm. **(F)** Corresponding intensity image of Ru-C_3_N_4_ in **(E)**. **(G)** XPS analysis of C_3_N_4_ and Ru-C_3_N_4_. **(H)** High-resolution XPS spectra of N 1s and **(I)** C 1s (or Ru 3d).

Four metal-C_3_N_4_ (the metal denoting Ru, Fe, Cu, and Ag) were prepared by heating the mixture of crystalline C_3_N_4_ and metal salts in an aqueous solution according to previous methods (Wang et al., 2019). As crystalline C_3_N_4_ provided strong coordination sites for metal ions, the metal can be effectively incorporated into this carrier. The morphologies of these metal-C_3_N_4_ and free C_3_N_4_ were investigated by transmission electron microscopy (TEM). As shown in Figure 1A and Supplementary Figure S1A–D, both the metal-C_3_N_4_ and free C_3_N_4_ exhibited very similar rod-like structure, indicating that there was no substantial morphological alteration following the integration of four metal ions.

The Ru-C_3_N_4_ was thoroughly studied due to its superior catalytic activity and antibacterial effect compared to other metal-C_3_N_4_, as demonstrated by our subsequent analysis. Scanning electron microscope (SEM) measurement clearly displayed the rod-like morphology of Ru-C_3_N_4_ (Figure 1B). Energy dispersive spectroscopy (EDS) mapping image revealed that Ru, C and N were uniformly distributed throughout the entire C_3_N_4_ architecture (Figure 1C; Supplementary Figure S2). For comparison, EDS mapping images of other metal-C_3_N_4_ were showed in Supplementary Figures S3A–C. Specifically, the concentrations of metal atoms anchoring on C_3_N_4_ were described as Ru (1.12 wt%), Fe (1.88 wt%), Cu (0.95 wt%) and Ag (0.66 wt%) quantified by inductively coupled plasma optical emission spectrometry (ICP-OES) (Supplementary Table S1). Additionally, high-angle annular dark-field image-scanning transmission electron microscopy (HAADF-STEM) measurements were conducted to provide the atomic information of metal-C_3_N_4_ (Figures 1D–F; Supplementary Figure S4). Figures 1D–F clearly showed single Ru atoms fixing across the C_3_N_4_ carrier, suggesting that no metal nanoparticle was formed during the heating of the synthesis process. Similarly, the HAADF-STEM images of other metal-C_3_N_4_ (Supplementary Figure S3) revealed that the metals were atomically dispersed on C_3_N_4_.

Next, the chemical composition and oxidation state of Ru-C_3_N_4_ were studied by X-ray photoelectron spectroscopy (XPS). The survey spectra of Ru-C_3_N_4_ and free C_3_N_4_ showed two peaks with binding energy around 287 eV and 398 eV, which belonged to C 1s (or Ru 3d) and N 1s, respectively (Figure 1G). The high-resolution N 1s spectra of Ru-C_3_N_4_ and free C_3_N_4_ were shown in Figure 1H. The N1s spectrum of free C_3_N_4_ was deconvoluted into two subpeaks centered at 398.15 and 399.69 eV, corresponding to sp^2^-hybridized pyridinic nitrogen (C-N=C) and sp^3^-hybridized tertiary nitrogen (N-(C)_3_), respectively. By contrast, the binding energy peak of C-N=C was observed at 398.32 eV, and the blue shift by 0.17 eV could be attributed to the electron transfer from nitrogen atoms of C_3_N_4_ to Ru ions for Ru-C_3_N_4_. Furthermore, Figure 1I provided the C 1s (or Ru 3d) spectra of Ru-C_3_N_4_ and free C_3_N_4_. The peaks at 284.60 and 287.84 eV in the C 1s spectrum of free C_3_N_4_ were attributed to the N-C=N bound and C-C bound, respectively. Notably, the peak around 284 eV in the C 1s spectrum of Ru-C_3_N_4_ was enhanced compared with free C_3_N_4_ due to the overlap of the C 1s and Ru 3d_3/2_ peaks (Chen et al., 2018). Moreover, the Ru 3d spectrum was deconvoluted into two subpeaks at 281.84 eV and 285.63 eV, which were attributed to Ru 3d_5/2_ and Ru 3d_3/2_, respectively, suggesting that Ru atoms in Ru-C_3_N_4_ were present in the Ru^2+^ oxidation state.

### 2.2 Peroxidase-like activity evaluation of metal-C_3_N_4_

**FIGURE 2:**
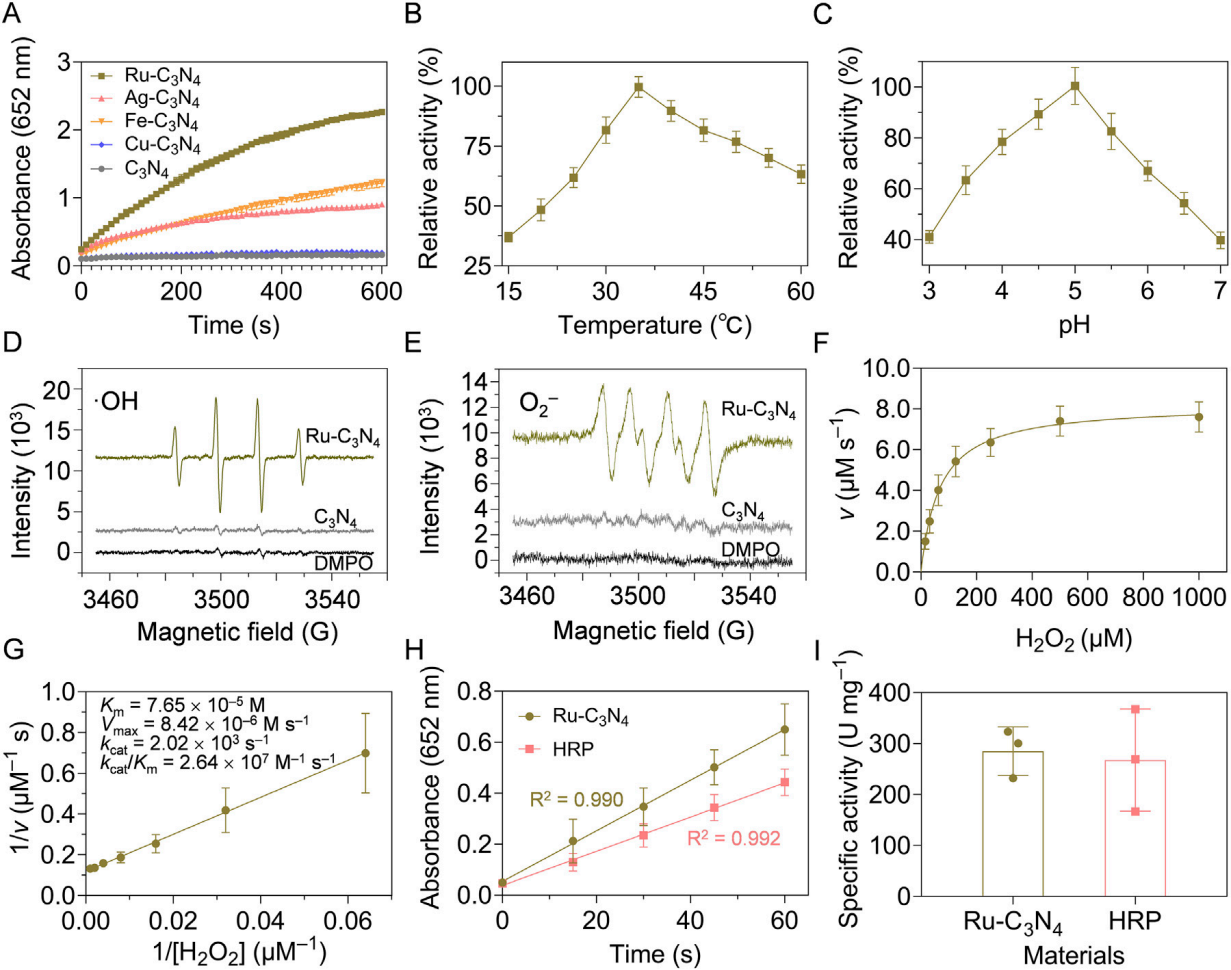
Peroxidase-like activity characterization of metal-C_3_N_4_. **(A)** Reaction-time curves of the TMB colorimetric reaction catalyzed by metal-C_3_N_4_. **(B)** The optimal temperature and **(C)** pH value of Ru-C_3_N_4_ for the catalytic activity. **(D)** ESR spectra showed the generation of **·**OH and **(E) ·**O_2_^−^ by Ru-C_3_N_4_. **(F,G)** Michaelis-Menten curves and kinetics parameters for Ru-C_3_N_4_. **(H,I)** The specific activities (U mg^−1^) of Ru-C_3_N_4_ and HRP.

ROS generation is a mainstream mechanism for the antibacterial activity of nanozymes (Song et al., 2022; Xu et al., 2023). Therefore, we next evaluated the peroxidase-like activity of four metal-C_3_N_4_ by catalyzing the generation of free radicals from H_2_O_2_ to achieve the oxidation of 3,3′,5,5′-tetramethylbenzidine (TMB). As shown in Figure 2A, Ru-C_3_N_4_ showed robust peroxidase-like activity, while the catalytic activity of Ag-C_3_N_4_ was close to that of Fe-C_3_N_4_, and both were weaker than that of Ru-C_3_N_4_. Conversely, the Cu-C_3_N_4_ and C_3_N_4_ carrier demonstrated no peroxidase-like activity, suggesting that the catalytic activity of metal-C_3_N_4_ resulted from their single-atom metals. For the color reaction, we employed different concentrations of Ru-C_3_N_4_ to further confirm its catalytic properties. Supplementary Figure S5 showed that the ability of Ru-C_3_N_4_ to catalyze the color reaction of TMB increased with its concentration.

The peroxidase-like activity of Ru-C_3_N_4_ was systematically analyzed under different reaction temperatures and pH values. With the increase of temperature (15°C–60°C), the catalytic activity of Ru-C_3_N_4_ showed a trend of increasing and then decreasing. Its optimal reaction temperature was close to 35°C, which was comparable with that of horseradish peroxidase (HRP) (Figure 2B; Supplementary Figure S6A). Further, Ru-C_3_N_4_ could maintain higher relative activity over a wider temperature range compared with HRP, indicating that Ru-C_3_N_4_ had greater potential for medical applications than the natural enzyme. Figure 2C and Supplementary Figure S6B exhibited that the optimal reaction pH values for Ru-C_3_N_4_ and HRP were 5.0 and 4.5, respectively. Under this condition, we evaluated Ru-C_3_N_4_ as a SANzyme-based antimicrobial to generate ROS in the presence of H_2_O_2_. The results of electron spin resonance (ESR) assays showed that Ru-C_3_N_4_ induced a strong intensity of two ROS, including **·**OH and**·**O_2_^−^. In contrast, its C_3_N_4_ carrier failed to catalyze H_2_O_2_ to produce ROS (Figures 2D, E).

The steady-state kinetic analyses of Ru-C_3_N_4_ and HRP were next compared comprehensively. Figure 2F and Supplementary Figure S7A demonstrated that the catalytic curves of Ru-C_3_N_4_ and HRP with H_2_O_2_ as the reaction substrate and TMB as the oxidation substrate followed the Michaelis-Menten equation. Figure 2G and Supplementary Figure S7B showed that the *K*_M_ value of Ru-C_3_N_4_ was 7.65 × 10^−5^ M, which was smaller than that of HRP (9.42 × 10^−5^ M) under the optimal reaction conditions, suggesting that the affinity of Ru-C_3_N_4_ for H_2_O_2_ was higher than that of HRP. Furthermore, other kinetics parameters of Ru-C_3_N_4_ and HRP, including *V*_max_, *k*_cat_, and *k*_cat_/*K*_M_, showed similar levels (Supplementary Table S2). To further characterize the enzymatic properties of Ru-C_3_N_4_, we calculated its peroxidase-like specific activity. The calculated protocols following the previous protocols (Jiang et al., 2018; Wang et al., 2024a). A length of 60 s was chosen for the initial rate period because the *R* ^2^ coefficient was close to 1.0 during this period after a linear-regression analysis (Figure 2H). As shown in Figure 2I, the mean peroxidase-like specific activity of Ru-C_3_N_4_ was 285.3 U mg^–1^, which was comparable to that of HRP (267.7 U mg^–1^). The outstanding peroxidase-like property of Ru-C_3_N_4_ SANzyme aligned with the previously reported enzymatic characteristics of Ru cluster nanozyme (Fan et al., 2024), indicating that Ru element is a promising material for the construction of high peroxidase-like activity nanozymes. In this work, we loaded single atomic Ru onto a crystalline C_3_N_4_, thereby enhancing its biosafety and contact area with bacteria. Additionally, the storage stability is an essential condition as antibacterial agents. Therefore, we finally investigated the catalytic stability of Ru-C_3_N_4_ under room temperature. Supplementary Figure S8 showed that there was no significant change in the peroxidase-like activity of Ru-C_3_N_4_ after the storage at room temperature for 20 weeks. Collectively, these data provide reliable evidence that Ru-C_3_N_4_ has excellent peroxidase-like activity.

### 2.3 Evaluation of inhibition of *S. aureus* growth by metal-C_3_N_4_ *in vitro*

**FIGURE 3:**
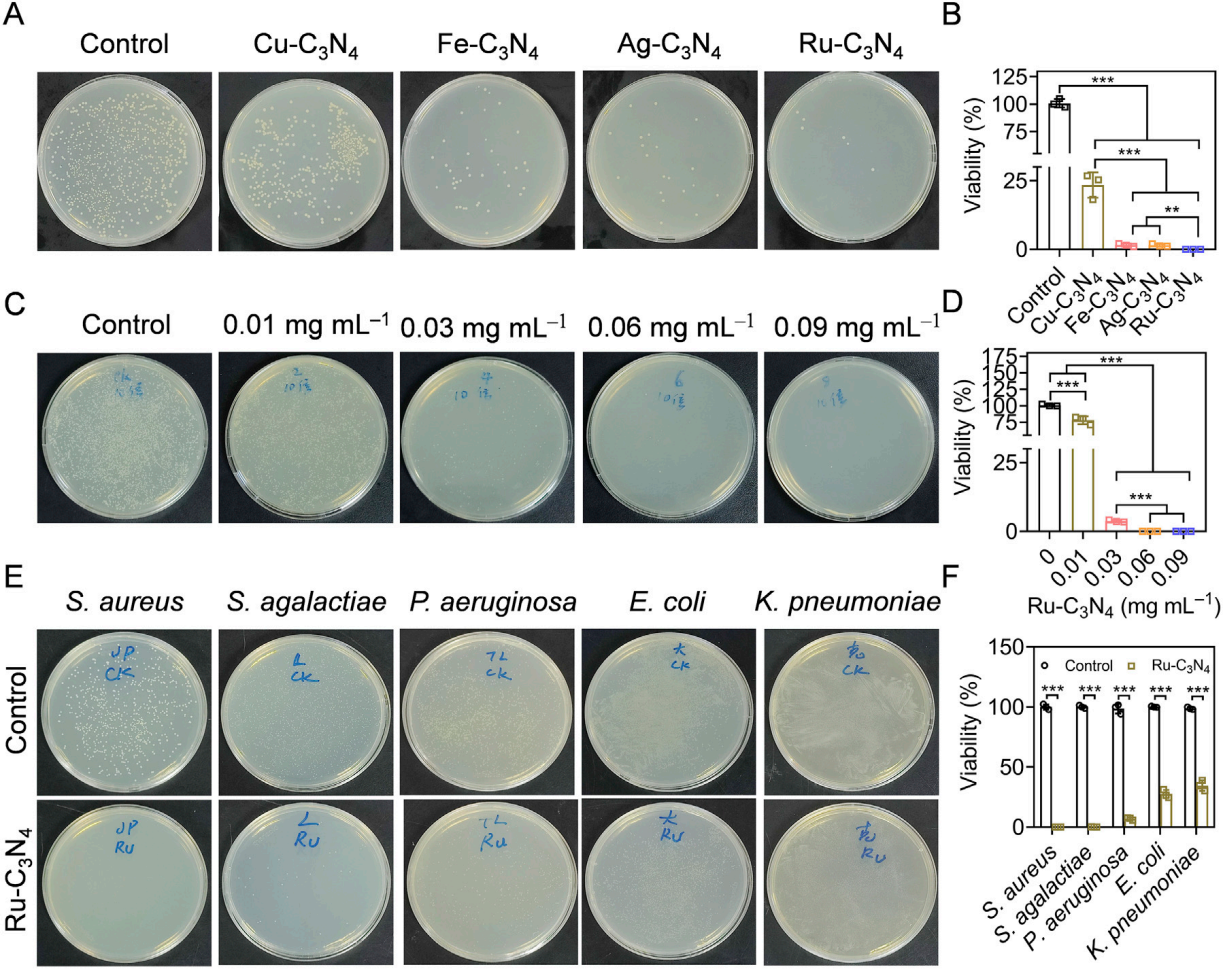
Evaluating the anti-*S. aureus* efficiency of metal-C_3_N_4_ *in vitro*. **(A,B)** The inhibition efficiency and the statistics of metal-C_3_N_4_ on *S. aureus* growth. Control represented only 10 mM H_2_O_2_ treatment, while other groups were treated with 10 mM H_2_O_2_ + metal-C_3_N_4_. **(C,D)** Anti-*S. aureus* efficiency the statistics of Ru-C_3_N_4_ at different concentrations (0.01–0.09 mg mL^–1^). Control represented only 10 mM H_2_O_2_ treatment, while other groups were treated with 10 mM H_2_O_2_ + 0.01–0.09 mg mL^–1^ Ru-C_3_N_4_. **(E,F)** Antibacterial efficiency and statistics of Ru-C_3_N_4_ against Gram-positive (*S. aureus*, *S. agalactiae*, and *P. aeruginosa*) and Gram-negative bacteria (*E. coli* and *K. pneumoniae*). Control represented only treated with 10 mM H_2_O_2_ for Gram-positive bacteria and 1 mM H_2_O_2_ for Gram-negative bacteria.

Catalytic activity studies have shown that Ru-C_3_N_4_ possessed stronger peroxidase-like activity than other metal-C_3_N_4_. However, which metal-C_3_N_4_ possesses the best antibacterial properties was unclear. To address this, we initially evaluated the inhibitory effects of four metal-C_3_N_4_ on *S. aureus* growth *in vitro*. As shown in Figure 3A, four metal-C_3_N_4_ exhibited different anti-*S. aureus* activities. Compared to control group, the bactericidal efficiency of Ru-C_3_N_4_ reached more than 99.9% when the concentration was 0.06 mg mL^–1^ and the incubation time was 0.5 h (Figure 3B). Under the same condition, other metal-C_3_N_4_ also showed significant antibacterial activity compared to control group. The antibacterial efficiency was Cu-C_3_N_4_ (76.6%), Fe-C_3_N_4_ (98.4%), and Ag-C_3_N_4_ (98.5%) respectively, which was largely positively correlated with their peroxidase-like activity. As a result, Ru-C_3_N_4_ was selected for further investigation.

Next, we optimized the administrated dosage (0.01–0.09 mg mL^–1^) of Ru-C_3_N_4_ to better understand its antibacterial effect. The results showed that the bactericidal effect of Ru-C_3_N_4_ progressively enhanced with increasing concentration (Figure 3C). The anti-*S. aureus* efficiency of Ru-C_3_N_4_ at 0.01, 0.03, 0.06, and 0.09 mg mL^–1^ was 25.1%, 96.3%, over 99.9%, and over 99.9%, respectively (Figure 3D). Therefore, we considered that 0.06 mg mL^–1^ was the optimal concentration of Ru-C_3_N_4_ for anti-*S. aureus* investigation. According to many current studies, some nanozymes with peroxidase-like activity have been attempted for the killing of *S. aureus*. For instance, the Au@ZIF-8 presented enhanced peroxidase-like activity under near-infrared laser (NIR). Au@ZIF-8 achieved *S. aureus* clearance rate up to 97% (Ren et al., 2023). MoWS_2_ nanozyme could achieve efficient anti-*S. aureus* activity (98.84%) and biofilm clearance through hyperthermia and reactive oxygen species under NIR-II irradiation (Du et al., 2024). Liang et al. designed a NiCo_2_O_4_ nanozyme with self-adaptive hierarchical nanostructure that can capture bacteria of various morphotypes via the physico-mechanical interaction between the nanostructure and bacteria. 1 mg mL^−1^ NiCo_2_O_4_ nanozyme exhibited superior bacterial inhibition rates of >99.99% against *S. aureus* (Song et al., 2024a). Compared with these nanozymes, low dose of Ru-C_3_N_4_ (0.06 mg mL^–1^) could achieved satisfactory antibacterial effect (>99%).

The broad-spectrum antibiotics exhibited potent antimicrobial properties, their application presented several drawbacks, including the propensity to select multiple bacterial species and the propagation of resistance. Additionally, they may disrupt the host microbiome’s diversity (Melander et al., 2018). If the specific cause of the infection is identified, the utilization of narrow-spectrum antimicrobials had the capability to alleviate the certain challenges (Casadevall, 2009; Alm and Lahiri, 2020). Therefore, we finally evaluated the antibacterial selectivity of Ru-C_3_N_4_. Three Gram-positive bacteria (*S. aureus*, *Staphylococcus agalactiae*, and *Pseudomonas aeruginosa*) and two Gram-negative bacteria (*E. coli* and *K. pneumoniae*) were cultured and treated with Ru-C_3_N_4_ at 0.06 mg mL^–1^. As shown in Figures 3E, F, Ru-C_3_N_4_ showed significant antibacterial effects against all strains. However, the killing effect of Ru-C_3_N_4_ on Gram-positive bacteria was significantly higher than that on Gram-negative bacteria (Supplementary Figure S9). The antibacterial efficiency of Ru-C_3_N_4_ against *S. aureus*, *S. agalactiae*, and *P. aeruginosa* was over 99.9%, 99.7%, and 93.0%, while that against *E. coli* and *K. pneumoniae* was 72.5% and 65.5%, respectively. Hence, Ru-C_3_N_4_ is relatively selective against Gram-positive bacteria.

### 2.4 Ru-C_3_N_4_ disrupts *S. aureus*-formed biofilms

**FIGURE 4:**
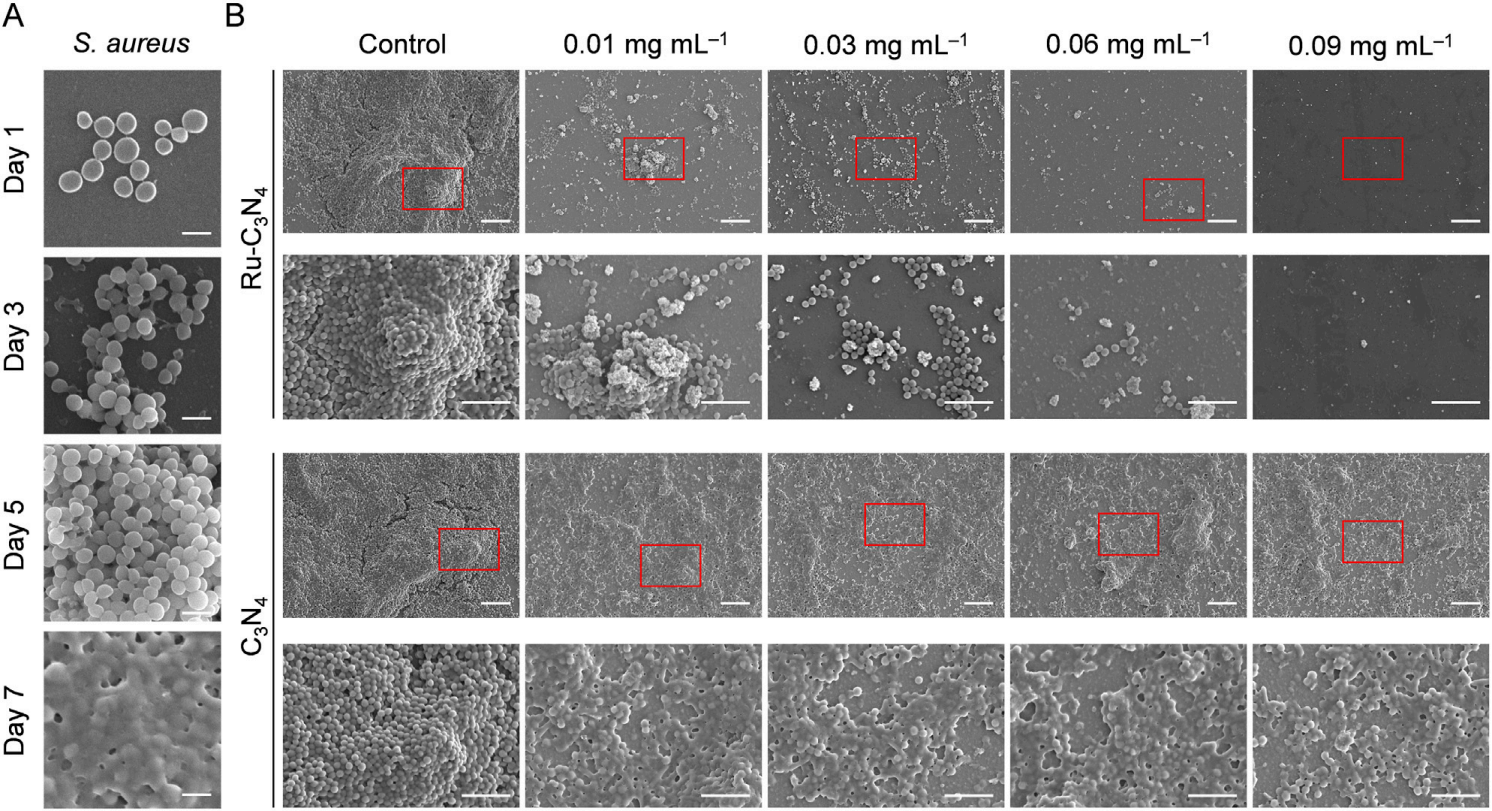
The destructive effect of Ru-C_3_N_4_ on the biofilms formed by *S. aureus*. **(A)** The process of biofilm formation by *S. aureus* in 1–7 days. Scale bar = 1 μm. **(B)** Destructive effects of Ru-C_3_N_4_ at different concentrations on *S. aureus*-formed biofilms. Control represented only 10 mM H_2_O_2_ treatment, while other groups were treated with 10 mM H_2_O_2_ + 0.01–0.09 mg mL^–1^ Ru-C_3_N_4_ or Ru-C_3_N_4_ carrier. The scale bars for the upper and lower parts of Ru-C_3_N_4_ and C_3_N_4_ treated groups are 10 μm and 5 μm, respectively.

Biofilm formation is a survival strategy for bacteria to adapt to their living environment, especially in the hostile environment (Wu et al., 2015). Bacterial biofilms play a critical role in the development of numerous persistent diseases (Mah, 2012). The structure and physiological characteristics of biofilms provide them with a natural resistance to antimicrobial agents (Donlan and Costerton, 2002). Once the biofilms become incomplete, bacteria will lose their shield and return to their planktonic state, becoming more sensitive to antibacterial agents (da Silva et al., 2021; Goodman and Bakaletz, 2022; Ji et al., 2022). Thus, we systematically evaluated the disrupted effect of Ru-C_3_N_4_ on *S. aureus*-formed biofilms. As shown in Figure 4A, *S. aureus* attached themselves to the surface of cell crawling sheets by using their sticky appendages, which resulted in the formation of microaggregation. Initially, filamentous junctions were formed between bacteria during biofilms formation. With the increase of culture time, bacteria-secreted polysaccharide matrix, fibrin, lipid protein, and other substances formed a bacteria gathered membrane-like substance. We then compared the effect of Ru-C_3_N_4_ at different concentrations (0.01–0.09 mg mL^–1^) on the destruction of biofilms. Figure 4B exhibited that C_3_N_4_ carrier was unable to cause disruption of *S. aureus*-formed biofilms. By contrast, Ru-C_3_N_4_ at 0.01 mg mL^–1^ could cause biofilm destruction moderately. With the increase of Ru-C_3_N_4_ concentration, the destruction effect of Ru-C_3_N_4_ on biofilms became more significant. 0.03 mg mL^–1^ Ru-C_3_N_4_ induced obvious biofilm destruction. *S. aureus* lost their shield and returned to planktonic state. Ru-C_3_N_4_ at 0.09 mg mL^–1^ achieved complete elimination of *S. aureus* and its biofilms. Therefore, the single atom Ru on Ru-C_3_N_4_ determines its antimicrobial activity. In the bacterial microenvironment, Ru-C_3_N_4_ first catalyzed H_2_O_2_ to produce large amounts of ROS. These free radicals effectively destroyed *S. aureus*-formed biofilms. Finally, the growth and structure of planktonic *S. aureus* were severely disrupted under excessive ROS condition.

### 2.5 Evaluation of anti-*S. aureus* effect of Ru-C_3_N_4_ *in vivo*

**FIGURE 5:**
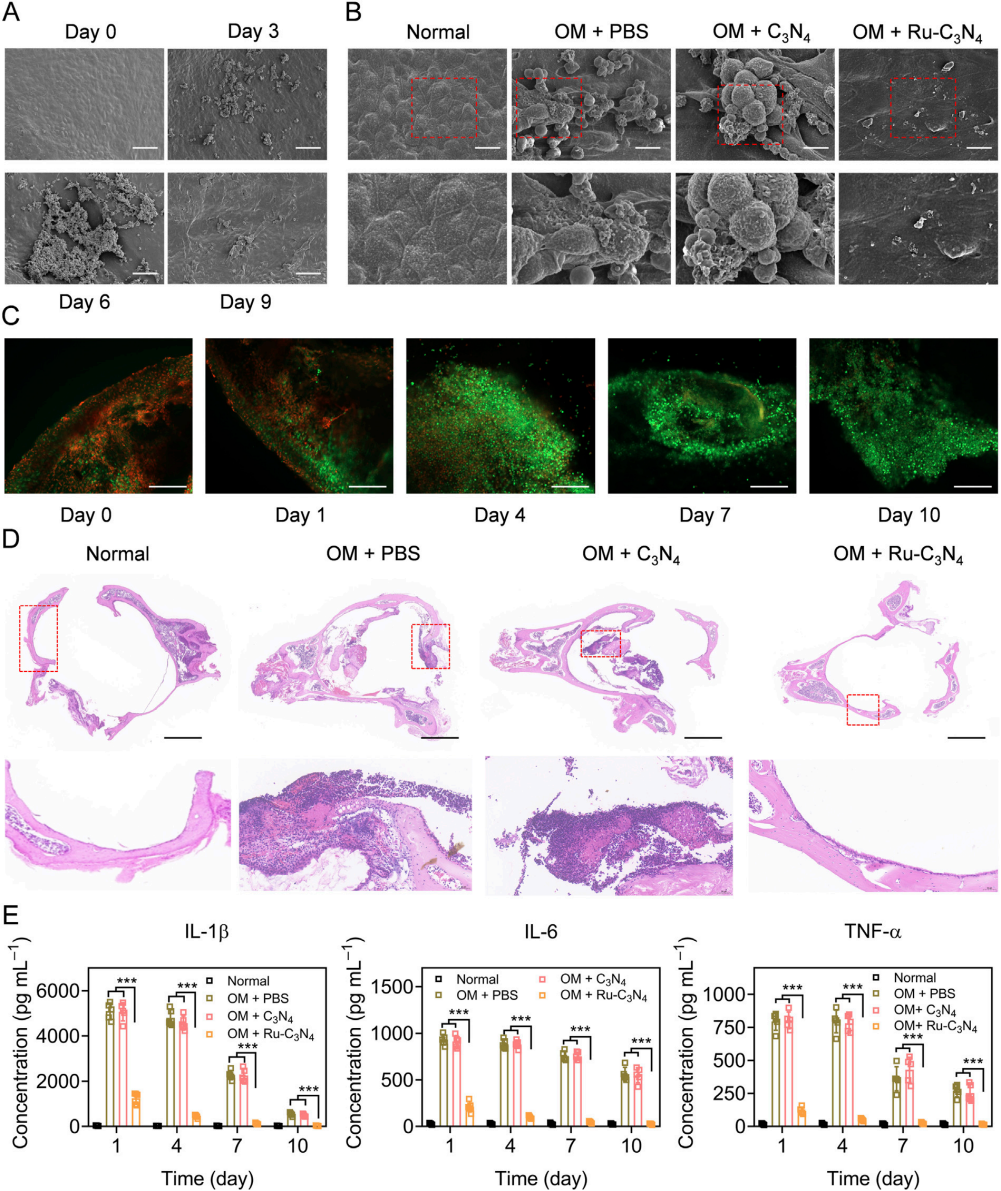
Anti-*S. aureus* effect of Ru-C_3_N_4_ in OM model. **(A)** The growth and biofilm formation of *S. aureus* in middle ear mucosa of C57BL/6 mouse. The scale bar = 50 μm. **(B)** Ru-C_3_N_4_ effectively disrupted the growth and biofilm formation of *S. aureus in vivo*. The scale bar = 10 μm. **(C)** Live/dead cells staining for the middle ear mucosa of OM model. The scale bar = 100 μm. **(D)** H&E staining for the middle ear. The scale bar = 1 mm. **(E)** Inflammatory cytokines detection in the middle ear lavage fluid of OM model.

We next investigated the anti-*S. aureus* effect of Ru-C_3_N_4_ *in vivo*. The OM model was constructed using C57BL/6J mice according to previous studies (Davidoss et al., 2018; Li et al., 2023). The histological morphology and infected process of the middle ear mucosa were observed by SEM system at different time points (0–9 days). As shown in Figure 5A, the middle ear mucosa of OM mouse model presented a typical physiological morphology at day 0. By contrast, many small bacterial colonies of *S. aureus* were found on the surface of middle ear mucosa at day 3. At day 6, *S. aureus* significantly formed interbacterial plasmodesmata and secreted abundant substances. Furthermore, *S. aureus* formed thick biofilms on the surface of middle ear mucosa after 9 days modelling, with the entire surface being completely covered, suggesting the successful construction of OM model. Subsequently, we evaluated the destruction effects of Ru-C_3_N_4_ for biofilms *in vivo*. Figure 5B showed that C_3_N_4_ carrier failed to inhibit the biofilm formation of *S. aureus*. On the contrary, Ru-C_3_N_4_ exhibited effective biofilm destruction and *S. aureus* killing effects after 7 days of treatment. Very few *S. aureus* were able to survive on the middle ear mucosa.

To investigate the therapeutic effect of Ru-C_3_N_4_ on OM model, the live/dead cell assay using calcein AM/propidium iodide (PI) staining was performed. At day 0 of Ru-C_3_N_4_ treatment, many dead cells (red) accumulated on the middle ear mucosa, indicating that the integrity of mucosal cells were severely damaged in OM model. With the extension of treatment time, the dead cells gradually disappeared and middle ear mucosal cells were fully recovered (green). At day 10 of Ru-C_3_N_4_ treatment, the dead mucosal cells of middle ear almost disappeared (Figure 5C), suggesting the mucosal destruction caused by *S. aureus* has been fully repaired. In addition, hematoxylin and eosin (H&E) staining was performed to further confirm the therapeutic effect of Ru-C_3_N_4_ on OM model. In the middle ear tissue of normal mouse, every type of cells was stained uniformly and packed tightly. Moreover, there was no obvious necrosis, bleeding, and inflammation in the ear tissues. The laminae propria connective tissue in normal ear tissues was equally distributed. As shown in the middle ear tissue of OM model, connective tissue hyperplasia was appeared, accompanied by eosinophilic exudate in the laminae propria. Epithelium exfoliation locally, new capillaries in the deep mucosa, and bone destruction were observed clearly. Meanwhile, several inflammatory features were present, including the infiltration of neutrophils and lymphocytes. These pathological features were not effectively improved in the C_3_N_4_ carrier treated group. By contrast, typical OM features of ear tissues almost disappeared after Ru-C_3_N_4_ treatment, accompanied by gradual healing of mucosal epithelium, alleviation of edema symptoms and inflammation, and repair of connective tissue (Figure 5D). Several inflammatory cytokines, including IL-1β, IL-6, and TNF-α, were obviously increased in the middle ear lavage fluid of OM model at day 0 (Supplementary Figure S10). Following the initial administration of Ru-C_3_N_4_, a significant decrease in the levels of all three inflammatory factors was observed. As the duration of treatment extended, their levels continued to decrease, eventually reverting to those in the normal group. However, C_3_N_4_ carrier had no significant effects on these inflammatory factors (Figure 5E).

### 2.6 Ru-C_3_N_4_ relieves *S. aureus*-induced hearing damage

**FIGURE 6:**
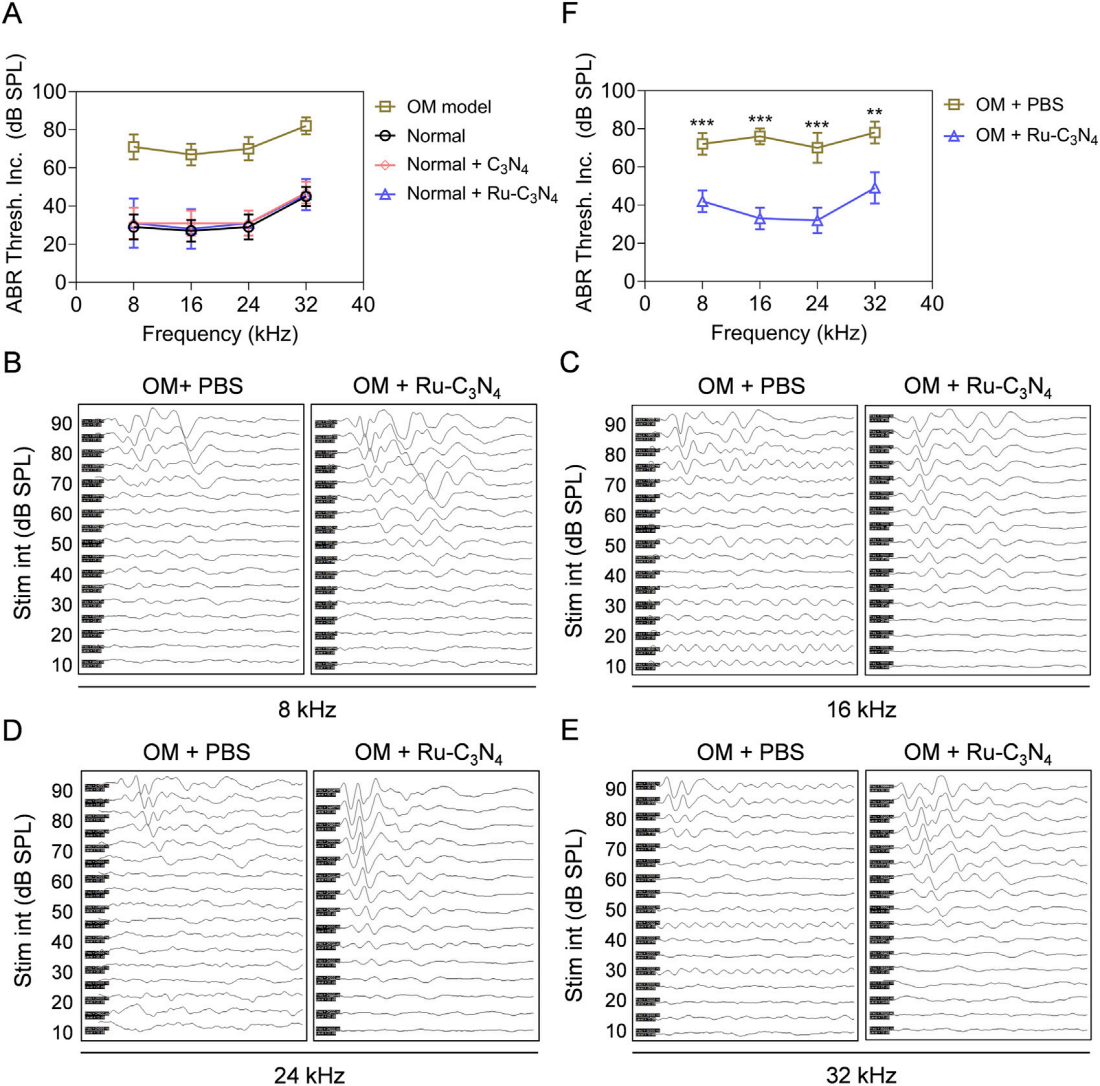
Ru-C_3_N_4_ relieves *S. aureus*-induced hearing damage. **(A)** C_3_N_4_ and Ru-C_3_N_4_ treatment did not affect the hearing function of normal mice. **(B–E)** Changes in hearing performance of OM mice after different treatments. **(F)** The statistics of ABR threshold for **(B–E)**.

With the excellent anti-*S. aureus* activity of Ru-C_3_N_4_ both *in vitro* and *in vivo*, we subsequently evaluated the efficacy of Ru-C_3_N_4_ in the treatment of hearing impairment caused by *S. aureus*. The auditory brainstem response (ABR) threshold of OM model was systematically tested. *In vivo*, the hearing function of normal mice under certain frequencies (8, 16, 24, 32 kHz) was not impaired by the treatments of Ru-C_3_N_4_ and C_3_N_4_ carrier (Figure 6A), suggesting the well biocompatibility of Ru-C_3_N_4_ SANzymes. By contrast, the ABR threshold of *S. aureus*-induced OM model was significantly decreased compared to normal mice, further suggesting the effectiveness of the established OM model.

We next conducted a systematic examination of the impact of Ru-C_3_N_4_ on auditory improvement in OM model. Figures 6B–E illustrated the ABR waveforms at frequencies of 8, 16, 24 and 32 kHz from both PBS- and Ru-C_3_N_4_-treated OM models. The ABR thresholds for the PBS-treated group were recorded as 70, 65, 65 and 70 dB at these frequencies, while those for the Ru-C_3_N_4_-treated group were found to be 35, 30, 20 and 40 dB. Compared with PBS-treated group, the average ABR thresholds at frequencies of 8, 16, 24 and 32 kHz decreased by amounts of 30, 43, 38 and 29 dB respectively in Ru-C_3_N_4_-treated group (Figure 6F). These results suggested that *S. aureus* infection caused a marked inflammatory response in the middle ear tympanic part. Inflammatory fluid limited the transmission of mechanical waves to the inner ear, resulting in conductive hearing loss. However, the structure and function of the hair cells were not affected. Therefore, *S. aureus*-induced hearing loss is temporary and reversible. After Ru-C_3_N_4_ treatment, *S. aureus* is effectively eliminated, which lead to the alleviation of inflammation in the middle ear and gradual restoration of hearing.

The biosafety of nanomedicines is critical to their further application. We finally examined the toxicity of Ru-C_3_N_4_ to cells and mice. We introduced two common human cell lines, named human umbilical vein endothelial cell (HUVEC) and human skin fibroblast cell (HSF), to evaluate the biosafety of Ru-C_3_N_4_ *in vitro*. The results of calcein AM/PI staining assay showed that Ru-C_3_N_4_ with 100–500 μg mL^–1^ did not cause significant cell death compared to PBS group (Supplementary Figure S11). *In vivo* evaluation, 10 mg kg^–1^ C_3_N_4_ or Ru-C_3_N_4_ was intravenously administered to normal mice on day 0, 4, 8, 12, 16, 20, 24, and 28. As shown in Supplementary Figure S12, the growth curves of C_3_N_4_- and Ru-C_3_N_4_-treated mice exhibited no obvious difference compared to that of PBS-treated group. We then determined the bleeding and clotting time to reflect the hematotoxicity of Ru-C_3_N_4_. Supplementary Figures S13A, B illustrated that the bleeding and clotting times for normal mice were approximately 80.49 s and 92.04 s, respectively. In contrast, the C_3_N_4_- and Ru-C_3_N_4_-treated mice exhibited bleeding time of 80.41 s and 80.45 s, and clotting time of 91.95 s and 92.03 s, respectively. To evaluate the effects of Ru-C_3_N_4_ on liver and kidney function, we tested the levels of relevant indicators in mouse blood. Following treatment with C_3_N_4_ or Ru-C_3_N_4_, there were no significant changes observed in the levels of alanine transaminase and aspartate aminotransferase (two liver injury indicators). Similarly, no significant alterations were noted in the levels of two kidney injury indicators, blood urea nitrogen and creatinine (Supplementary Figure S14). Therefore, Ru-C_3_N_4_ and its carrier exhibited high biosafety, which provides sufficient guarantee for its clinical applications.

## 3 Conclusion

In this study, four antibacterial metals (Cu, Fe, Ag, and Ru) were coordinated on a crystalline C_3_N_4_ carrier. The synthesized metal-C_3_N_4_ expanded the contact area with their surroundings, thereby enhancing its efficiency in binding to substrates and microorganisms. Among these metal-C_3_N_4_, Ru-C_3_N_4_ demonstrated the highest peroxidase-like activity. Utilizing its catalytic activity, Ru-C_3_N_4_ effectively inhibited *S. aureus* growth and destroyed *S. aureus*-formed biofilms, exhibiting robust anti-*S. aureus* efficiency (>99.9%). In the *S. aureus*-induced OM model, Ru-C_3_N_4_ successfully eliminated *S. aureus*, reducing inflammation in the ear microenvironment, and finally promoting the repair of middle ear mucosal cells and hearing recovery. Additionally, the low loading of Ru atoms on C_3_N_4_ carrier ensured the excellent biosafety of Ru-C_3_N_4_. In summary, this work provides valuable insights into the design of highly efficient antibacterial SANzymes. The selected Ru-C_3_N_4_ serves as an effective nanoantibiotic for treating *S. aureus*-induced OM.

## 4 Materials and methods

### 4.1 Preparation of C_3_N_4_

The C_3_N_4_ nano-crystals were achieved via an ionothermal synthesis approach according to our previous work (Wu et al., 2023). Typically, 2 g melamine were dispersed in 10 mL deionized water by vigorous agitation, whereafter ultrasonicated for 30 min. The mixture was subsequently refluxing for 3 h and dehydrated at 80°C for 8 h. The dried samples were ground and placed on the corundum porcelain boat of muffle furnace for 4 h of heating at 500°C with 5°C min^−1^ rate. After cooling, the sample was ground with a mixture of LiCl/KCl (molar ratio = 59: 41) in a mortar, followed by heating at 550°C for 4 h with 2.5°C min^−1^ rate under a N_2_ flow in the tubular furnace. After cooling to room temperature naturally, the resulting sample was washed with boiling deionized water to remove residual salt and air-dried at 60°C for 8 h.

### 4.2 Preparation of metal-C_3_N_4_

To synthesize Ru-C_3_N_4_, 10 mg of C_3_N_4_ were first dispersed into 10 mL of ultrapure water. Subsequently, 2 mL RuCl_3_ solution (2 mg mL^−1^) was added into the above solution, which was then refluxed at 100°C for 4 h. The Ru-C_3_N_4_ was obtained by centrifuging and washing with water twice to remove the excess RuCl_3_. Fe-C_3_N_4_, Cu-C_3_N_4_, Ag-C_3_N_4_ were prepared in a similar procedure where equivalent mass of FeCl_3_, CuCl_2_, or AgNO_3_ precursors were used instead of RuCl_3_. The temperature of reaction system was 80°C, 100°C, and 80°C, respectively.

### 4.3 The characterization of metal-C_3_N_4_

Transmission electron microscopy (TEM) was performed by a FEI-Talos F200X microscope at an acceleration voltage of 200 kV (FEI, United States). Scanning electron microscope (SEM) was carried out by GeminiSEM 300 at 5 keV (ZEISS, Germany). High-angle annular dark-field image-scanning transmission electron microscopy (HAADF-STEM) was conducted at 200 kV on a JEM ARM 200F instrument (JEOL, Japan). X-ray photoelectron spectroscopy (XPS) spectra were acquired on an Escalab 250Xi (Thermo Scientific, United States). Inductively coupled plasma optical emission spectrometry (ICP-OES) was performed on ICPOES730 (Agilent, United States).

### 4.4 The peroxidase-like activity of metal-C_3_N_4_

The peroxidase-like activity of SANzymes was described in our previous studies (Wang et al., 2024a; Wang et al., 2024b). In brief, the activity testing of the four metal-C_3_N_4_ was performed within a 100 μL system, including 10 μL TMB (2.08 mM), 10 μL H_2_O_2_ (0.5 M), 10 μL metal-C_3_N_4_ (2 μg mL^−1^), and 70 μL HAc-NaAc (0.02 M, pH = 4.5). The absorbance of oxTMB was recorded within a time-scan mode at 652 nm via SpectraMax M4 spectrophotometer (Molecular Devices, United States). Since Ru-C_3_N_4_ had the optimal peroxidase-like activity, we then tested its optimal temperature and pH value. The optimal temperature was tested within a 100 μL system containing 10 μL Ru-C_3_N_4_ (2 μg mL^−1^) or HRP (0.9 μg mL^−1^), 10 μL TMB (2.08 mM), 10 μL H_2_O_2_ (0.5 M) and 70 μL HAc-NaAc (0.02 M, pH = 4.5). The absorbance at 652 nm was recorded when the temperature ranged from 15°C to 60°C. The optimal pH of Ru-C_3_N_4_ was also detected in the same 100 μL system at 25°C when the pH value ranged from 3.0 to 7.0.

For the steady-state kinetic analysis, 100 μL reaction system containing 10 μL Ru-C_3_N_4_ (2 μg mL^−1^) or HRP (0.9 μg mL^−1^), 10 μL TMB (2.08 mM), 70 μL HAc-NaAc (pH = 5.0, 0.02 M), and a series of concentrations of H_2_O_2_ range from 0 to 1000 μM. The Michaelis-Menten constant was calculated using the following formula:

$$v=\left(v_{\max}\times \left[S\right]\right)/\left(K_{M}+\left[S\right]\right).$$

The catalytic constant ( $k_{cat}$ ) was calculated as follows:

$$k_{cat}=v_{\max}/\left[E\right]$$

where v represents the initial velocity, $K_{M}$ represents the Michaelis constant, $\left[S\right]$ represents the substrate concentration, and $v_{\max}$ represents the maximal reaction velocity.

The specific activities (U mg^−1^ Ru atoms) were calculated by the formula [*V*/(*Ɛ* × *Ɩ*) × (Δ*A*/Δ*t*)]/[*C*], where *V* denotes the total volume of reaction solution (μL); *Ɛ* denotes the molar absorption coefficient of the colorimetric substrate, which is typically maximized at 39,000 M^−1^ cm^−1^ at 652 nm for TMB. *Ɩ* denotes the path length of light traveling in the cuvette (cm); *A* denotes the absorbance after subtraction of the blank value; Δ*A*/Δ*t* denotes the initial rate of change in absorbance at 652 nm; and [*C*] denotes the concentration of HRP or Ru-C_3_N_4_ (mg).

### 4.5 Free radical identification

The generation of **·**OH and **·**O_2_^−^ was assessed through ESR analysis using dimethyl pyridine nitrogen oxide (DMPO) spin-trapping adduct. Glass capillary tubes with internal diameters of 1 mm were filled with 50 μL C_3_N_4_ or Ru-C_3_N_4_ and then sealed. These tubes were then inserted into the cavity of an ESR instrument (Bruker, Germany), with parameters set as follows: 1 G field modulation, 100 G scan range, and 20 mW microwave power. The spin trap DMPO was used to verify the formation of ROS during H_2_O_2_ degradation in presence of C_3_N_4_ or Ru-C_3_N_4_. Using the peak-to-peak height of the second line of the ESR spectra, the ROS production was estimated based on the signal intensity of the DMPO/·OH and DMPO/**·**O_2_^−^.

### 4.6 Bacterial culture and *in vitro* antibacterial activity

The *S. aureus* monoclonal was seeded to 5 mL of liquid LB medium and cultured for 8 h at 37°C and 180 rpm, respectively. When the bacterial solution reached the logarithmic growth cycle, it was diluted to 10^6^ colony-forming units (CFU) mL^–1^ for antibacterial testing. The *S. aureus* solution (10^6^ CFU mL^–1^) was treated with 1% PBS +10 mM H_2_O_2_ (Control), and different metal-C_3_N_4_ (0.06 mg mL^–1^) + 10 mM H_2_O_2_ at 37°C and 180 rpm for 0.5 h. Then the bacterial suspension was diluted with 1% PBS and distributed on solid medium and cultured at 37°C for 16 h to evaluate the antibacterial activity of metal-C_3_N_4_. To evaluate the optimal concentration of Ru-C_3_N_4_, we treated *S. aureus* with different concentrations of Ru-C_3_N_4_ (0, 0.01, 0.03, 0.06, 0.09 mg mL^–1^) and 10 mM H_2_O_2_ in NaAc buffer. The antibacterial efficiency of nanozymes was calculated as: (number of clones in experimental group/number of clones in control group) × 100%.

Next, three Gram-positive bacteria (*S. aureus*, *S. agalactiae*, and *P. aeruginosa*) and two Gram-negative bacteria (*E. coli* and *K. pneumoniae*) were used to evaluate the relative selectivity of Ru-C_3_N_4_. The concentration of H_2_O_2_ was 10 mM for Gram-positive bacteria and 1 mM for Gram-negative bacteria according to previous study (Xi et al., 2019). 0.06 mg mL^–1^ Ru-C_3_N_4_ + H_2_O_2_ were used to evaluate its antibacterial activity for the five bacteria. The detailed culture conditions were the same as described above.

### 4.7 *In vitro* destruction of biofilms

To investigate the destructive ability of Ru-C_3_N_4_ on biofilms, the bacterial suspension of *S. aureus* was cultured in the 24-well plate covered with cell crawling sheets. After 3–4 days of daily culture with fresh medium, a thin biofilm could be seen on the cell crawling sheets. Then different concentrations of Ru-C_3_N_4_ (0.01–0.09 mg mL^–1^) + 10 mM H_2_O_2_ were applied to treat the biofilms for 72 h. The destruction of biofilms was imaged by using S-4800 SEM system.

### 4.8 Biosafety analysis of Ru-C_3_N_4_

*In vitro*, HUVEC and HSF cells were cultured to 80% confluence and then treated with 1% PBS or 100–500 ug mL^–1^ Ru-C_3_N_4_ for 24 h. Next, the cells in ever group were stained with calcein AM/PI to distinguish live and dead cells. Finally, a laser scanning confocal microscope (LSM 800) was used to take pictures.

*In vivo*, C57BL/6J mice were intravenously administered a dose of 10 mg kg^–1^ C_3_N_4_ or Ru-C_3_N_4_ via the tail vein on day 0, 4, 8, 12, 16, 20, 24, and 28. Prior to each injection, body weight was meticulously measured. A scalpel was used to create a transverse incision over the lateral vein, following which the tail was immersed in 1% PBS at 37°C. The duration from the initiation of the incision until bleeding ceased was recorded as bleeding time. Blood samples were procured from each mouse’s orbital plexus using a glass capillary. The time required for fibrin threads to emerge between the fractured capillaries was documented as clotting time. The functional indexes of liver and kidney in mice, including alanine transaminase (ALT), aspartate aminotransferase (AST), blood urea nitrogen (BUN), and creatinine (CREA), were detected by their commercial ELISA kits.

### 4.9 OM model construction and therapy

All the animal procedures were approved by the Ethics Committee of Shenzhen Institutes of Advanced Technology, Chinese Academy of Sciences (Approval number: 20230727-A1682). The research adheres to globally accepted animal welfare standards, and all animal-related studies are also in compliance with the ARRIVE guidelines and the AVMA euthanasia guidelines 2020. The C57BL/6 J mice with 6–8 weeks old were utilized to construct OM model to evaluate the antibacterial efficacy of metal-C_3_N_4_ *in vivo*. All the mice underwent an otoscopic examination and an ABR test to confirm that their tympanic membranes were normal and no middle ear effusion was present. The external auditory canal of mouse was sterilized before modelling. The suspension of *S. aureus* (1.0 × 10^6^ CUF mL^–1^, 25 µL per ear) was injected through tympanic membrane puncture under sterile conditions. The OM mice were divided into three groups and injected with 1% PBS +10 mM H_2_O_2_, 0.2 mg mL^–1^ C_3_N_4_ + 10 mM H_2_O_2_, or 0.2 mg mL^–1^ Ru-C_3_N_4_ + 10 mM H_2_O_2_. The morphological changes of biofilms were evaluated with an S-4800 SEM system.

The tissues were collected and rinsed 3 times with 1% PBS after varied treatments. The samples were fixed with 2.5% glutaraldehyde overnight at 4°C. Subsequently, the samples were treated with an EDTA decalcifying solution (PH = 7.2) to soften the tissues in preparation for further testing. After 1% PBS washing, the samples were dehydrated with ethanol solutions of several concentrations (30%, 50%, 70%, 80%, 95% and 100%). Finally, the samples were dried in a desiccator for 3 h and fixed on the plate with conductive glue and then sprayed with gold for 1 min. The result was obtained with an S-4800 SEM system.

### 4.10 Live/dead cells staining assay

After different treatments, the middle ear mucosa of OM model was separated and stained with calcein AM/PI. Commercially available dyes calcein AM and PI were used to distinguish live and dead cells. Calcein AM could result in green fluorescence when staining live cells. PI is a membrane-impermeable red dye. Finally, the living and dead cells were imaged with a laser scanning confocal microscope LSM 800.

### 4.11 Histological staining

Typical H&E staining was used to observe the histomorphology of the ears. Briefly, the ear tissues were obtained from the normal mice and different treated OM models. 4% paraformaldehyde was used to fix the tissues. Then the tissues were embedded with paraffin for further study. 5 μm pathological sections were obtained and stained with H&E.

### 4.12 Detection of TNF-α, IL-1β, and IL-6 in middle ear lavage fluid

The middle ear lavage fluids were collected from the normal mice and different treated OM models. The concentrations of TNF-α, IL-1β, and IL-6 in middle ear lavage fluids were quantified by mouse TNF-α, IL-1β, and IL-6 highly sensitive ELISA kits, respectively. All the measurements were strictly performed following the procedures provided by manufacturers.

### 4.13 Statistical analysis

Results were presented as the mean ± SD, derived from a minimum of three independent experiments, unless otherwise specified. Statistical analysis was conducted using the paired Student’s t-test, while comparisons involving more than two groups were evaluated through ANOVA. Graph plotting was executed utilizing GraphPad Prism 8.0 software. A significance level of *p* < 0.05 was adopted for statistical significance. *p < 0.05, **p < 0.01, and ***p < 0.001.

## Data Availability

The original contributions presented in the study are included in the article/Supplementary Material, further inquiries can be directed to the corresponding authors.
