# Supplementary material for: A ruthenium single atom nanozyme-based antibiotic for the treatment of otitis media caused by Staphylococcus aureus
Source: Front Chem. 2024 Aug 28;12:1439039. doi: 10.3389/fchem.2024.1439039 (PMC11387182; doi:10.3389/fchem.2024.1439039)
Supplement: Supplementary file 1 [file DataSheet1.docx]

Supplementary Material

**A ruthenium single atom nanozyme-based antibiotic for the treatment of otitis media caused by *Staphylococcus aureus***

Jie Wang ^†1^, Rui Gong^†1,2^, Ming Yang^†3^, Xi Wu^1^, Ziwei Li^4^, Haibing Huang^1^, Xiyun Yan*^1,5,6^, Daji Wang*^1^

^1^ Nanozyme Synthesis Center, Key Laboratory of Quantitative Synthetic Biology, Shenzhen Institute of Synthetic Biology, Shenzhen Institutes of Advanced Technology, Chinese Academy of Sciences, Shenzhen, Guangdong, China.

^2^ Faculty of Synthetic Biology, Shenzhen Institute of Advanced Technology, Chinese Academy of Sciences, Shenzhen, Guangdong, China.

^3^ Department of Otolaryngology, Shenzhen People’s Hospital (The Second Clinical Medical College, Jinan University; The First Affiliated Hospital, Southern University of Science and Technology), Shenzhen, Guangdong, China.

^4^ Department of Clinical Laboratory, Shenshan Central Hospital, Sun Yat-sen Memorial Hospital, Sun Yat-sen University, Shanwei, Guangdong, China.

^5^ CAS Engineering Laboratory for Nanozyme, Key Laboratory of Biomacromolecules, Institute of Biophysics, Chinese Academy of Sciences, Beijing, China.

^6^ Nanozyme Laboratory in Zhongyuan, Henan Academy of Innovations in Medical Science, Zhengzhou, Henan, China.

^†^ **First authorship:** These authors share first authorship.

*** Correspondence:**

Xiyun Yan

yanxy@ibp.ac.cn

Daji Wang
dj.wang1@siat.ac.cn

# Supplementary Figures and Tables

## Supplementary Figures


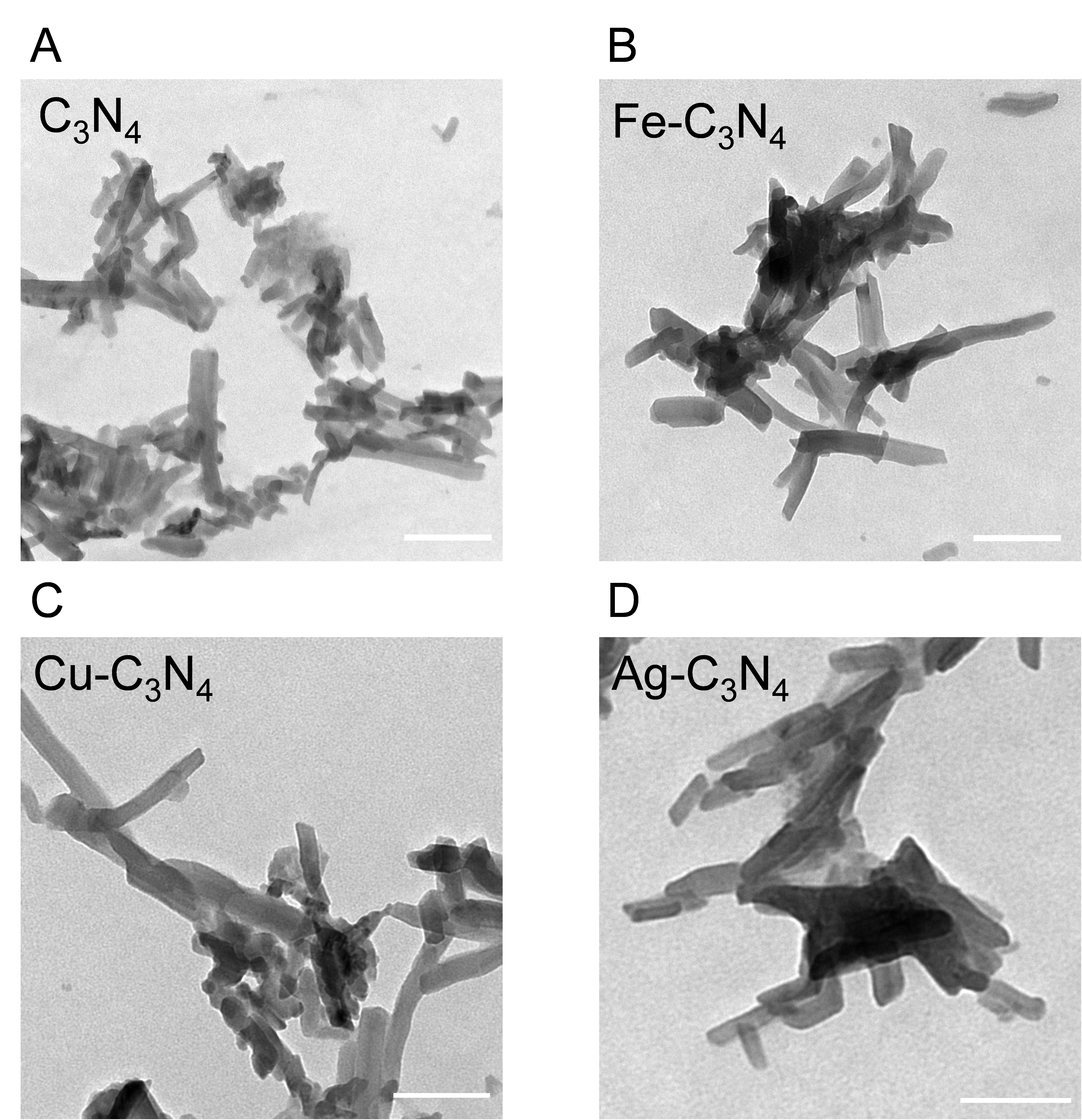


**Figure S1.** The TEM images of Fe-C_3_N_4_, Cu-C_3_N_4_, and Ag-C_3_N_4_. Scale bar = 100 nm.


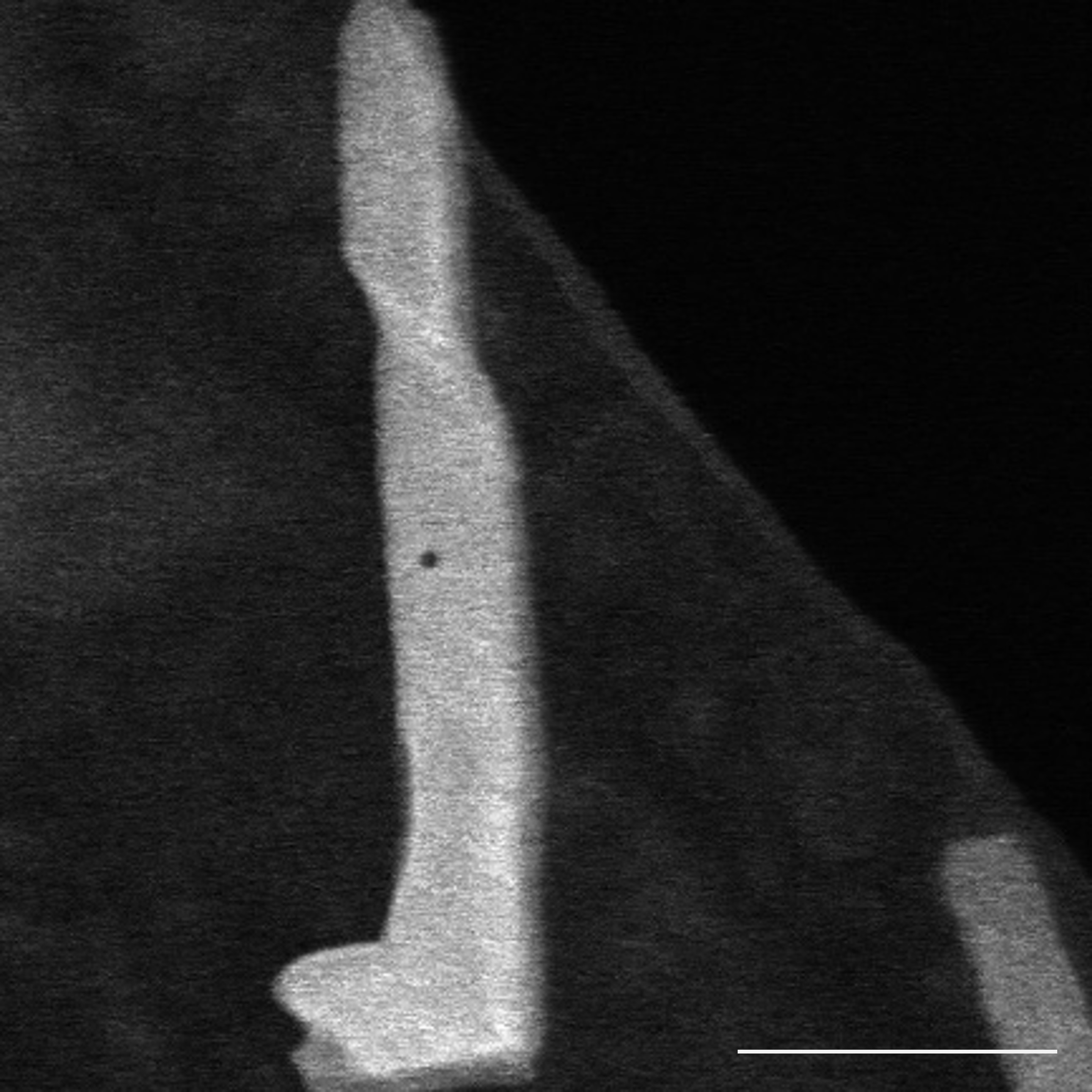


**Figure S2.** The bright EDS image of Ru-C_3_N_4_. Scale bar = 80 nm.


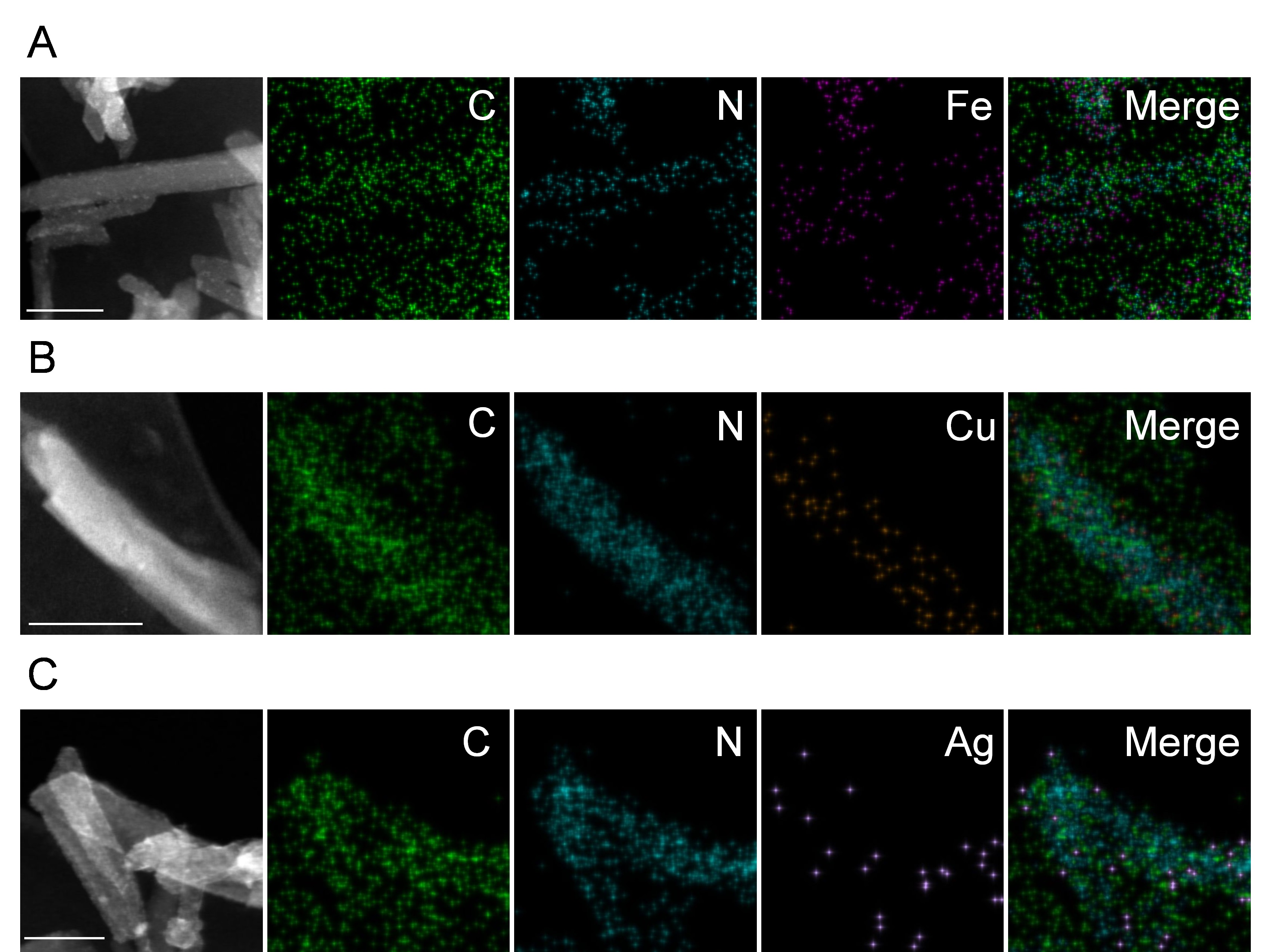


**Figure S3.** EDS mapping images of (A) Fe-C_3_N_4_, (B) Cu-C_3_N_4_, and (C) Ag-C_3_N_4_. Scale bar = 80 nm.


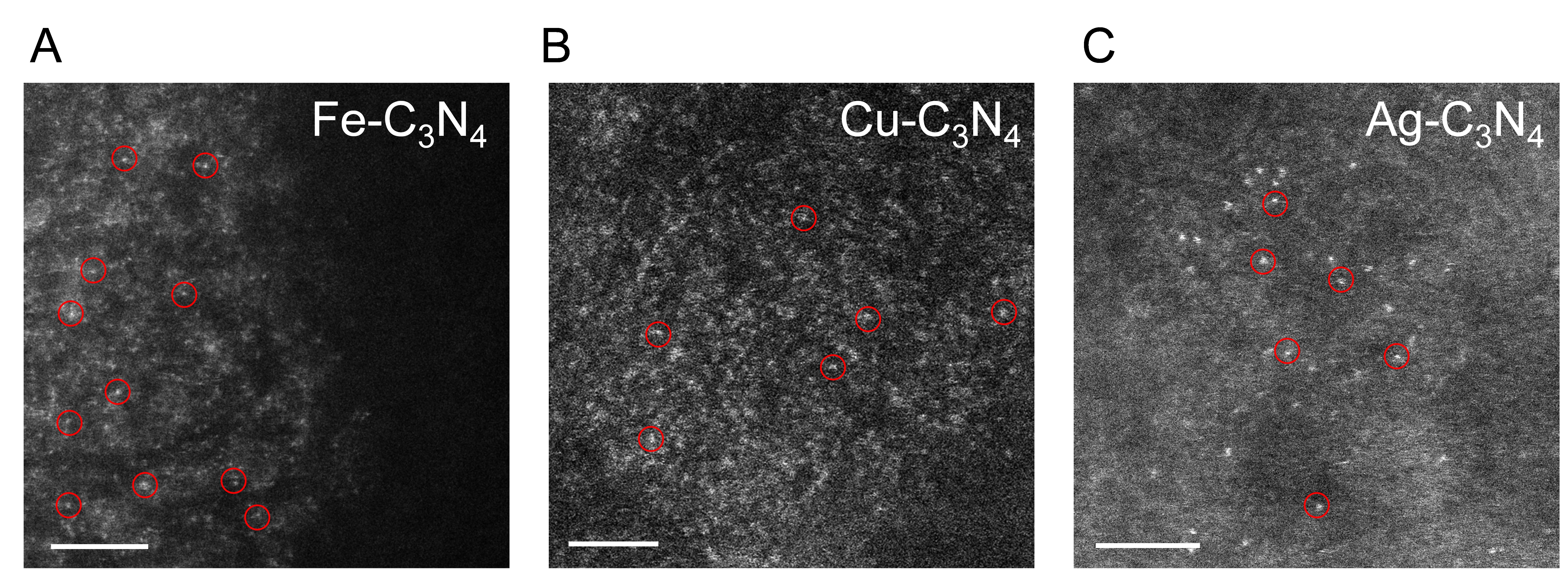


**Figure S4.** The HADDF-STEM images of Fe-C_3_N_4_, Cu-C_3_N_4_, and Ag-C_3_N_4_. Scale bar = 2 nm.


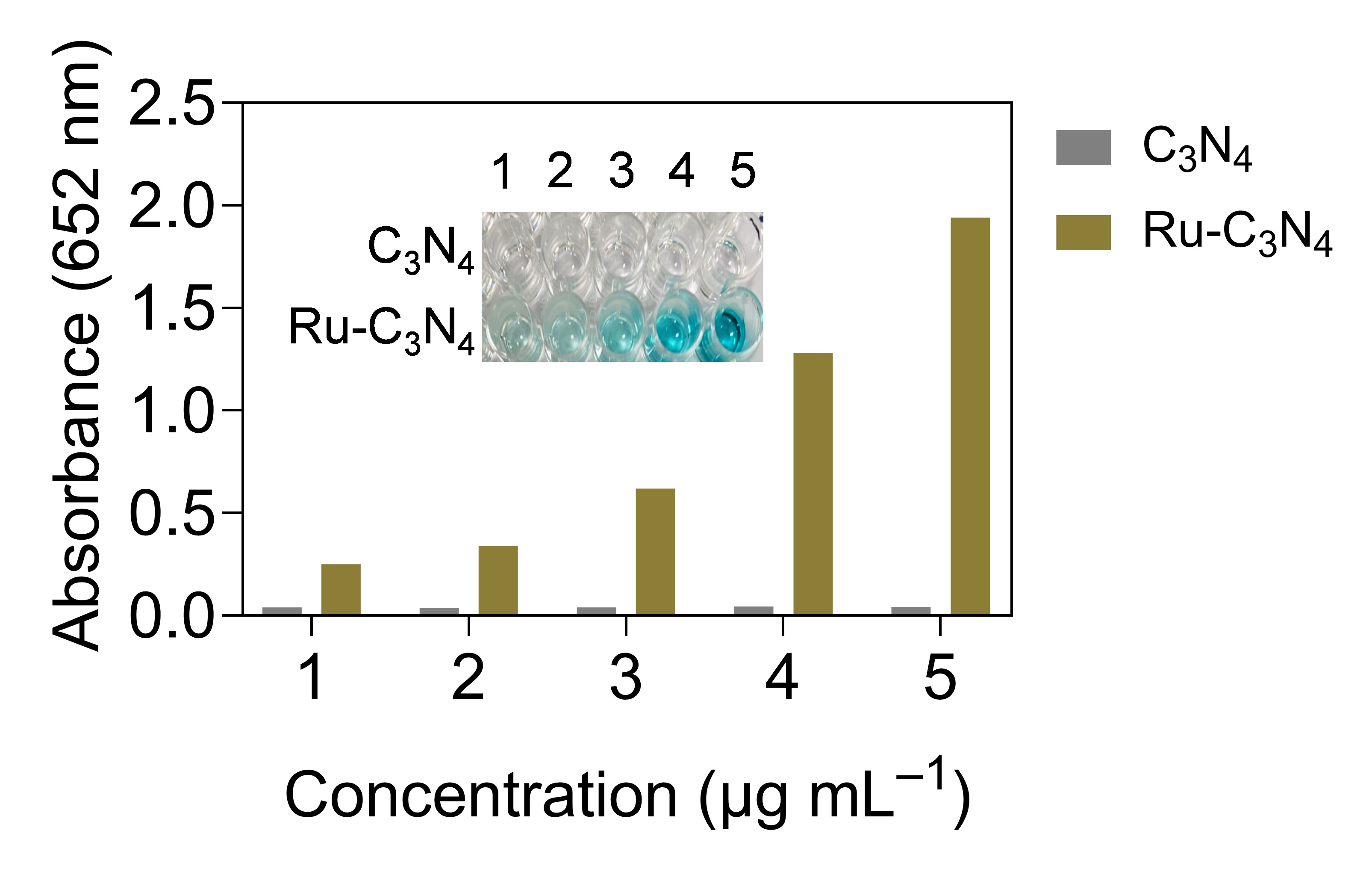


**Figure S5.** The color reaction of Ru-C_3_N_4_ with different concentrations.


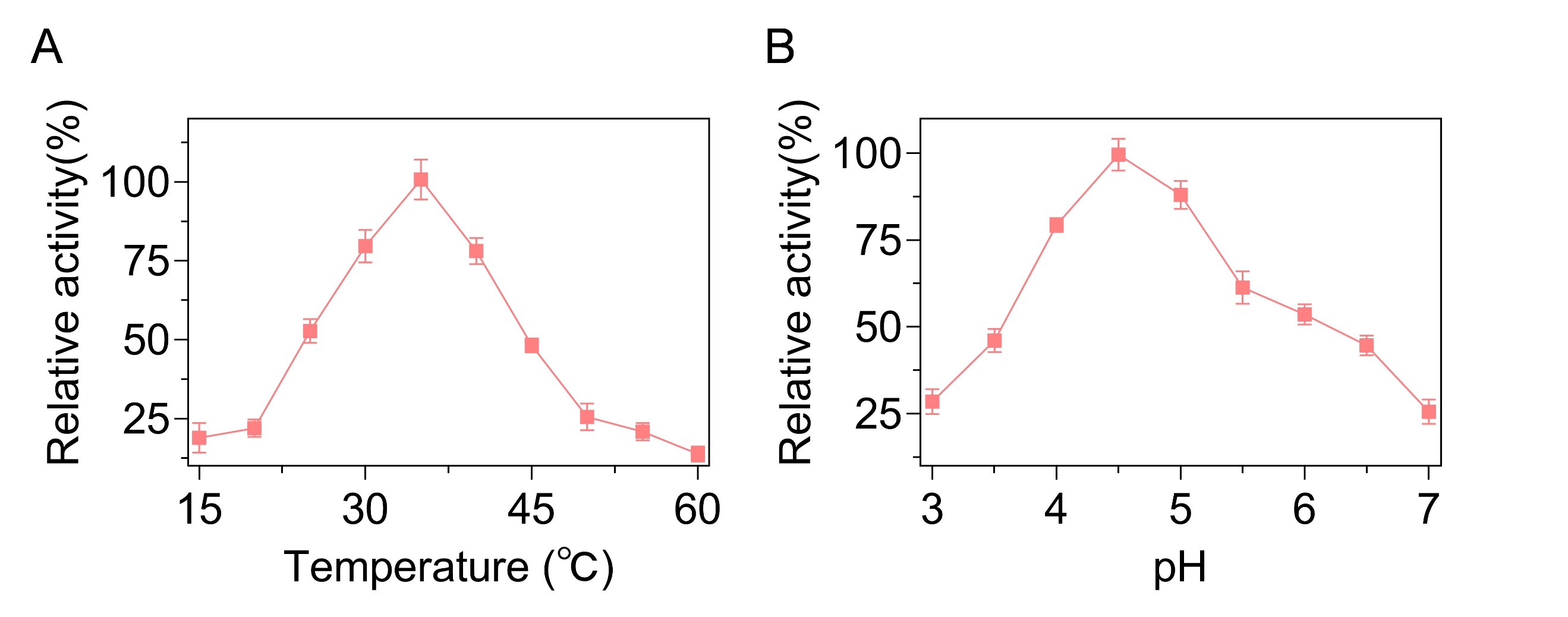


**Figure S6. (A)** The optimal temperature and **(B)** pH value of HRP for the catalytic activity.


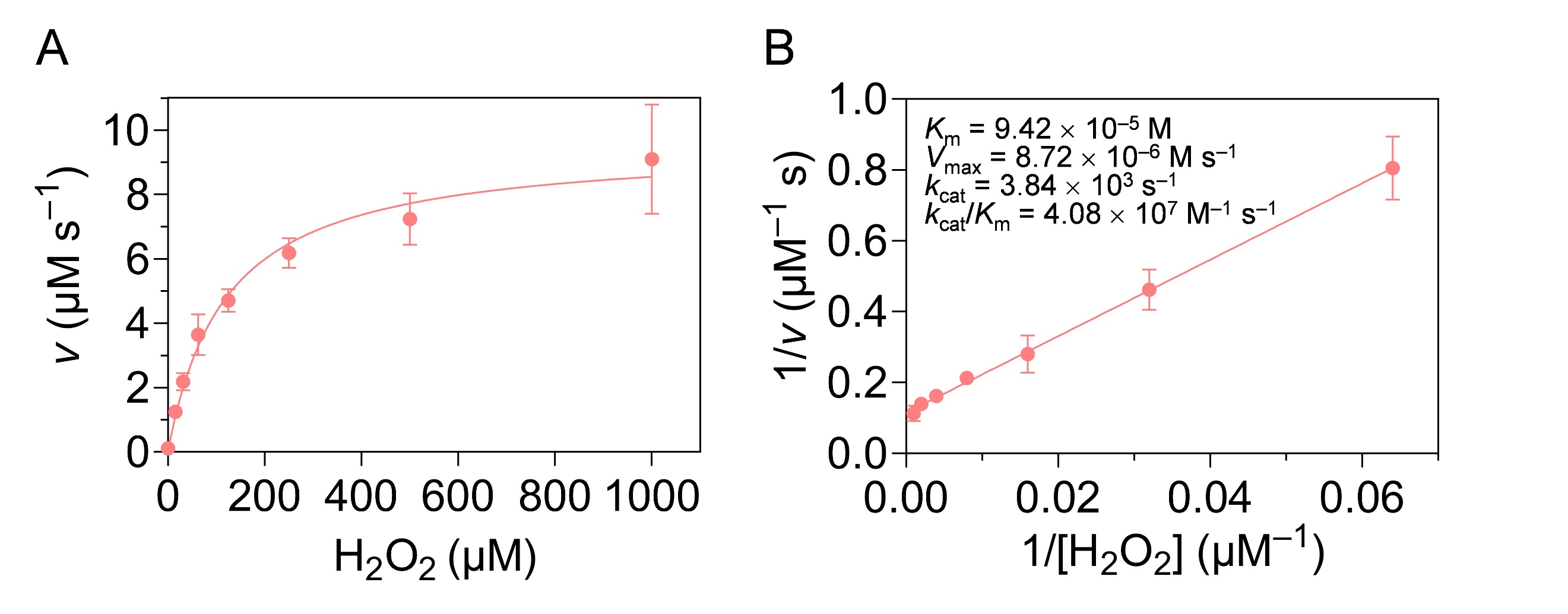


**Figure S7. (A-B)** Michaelis-Menten curve and kinetics parameters for HRP.


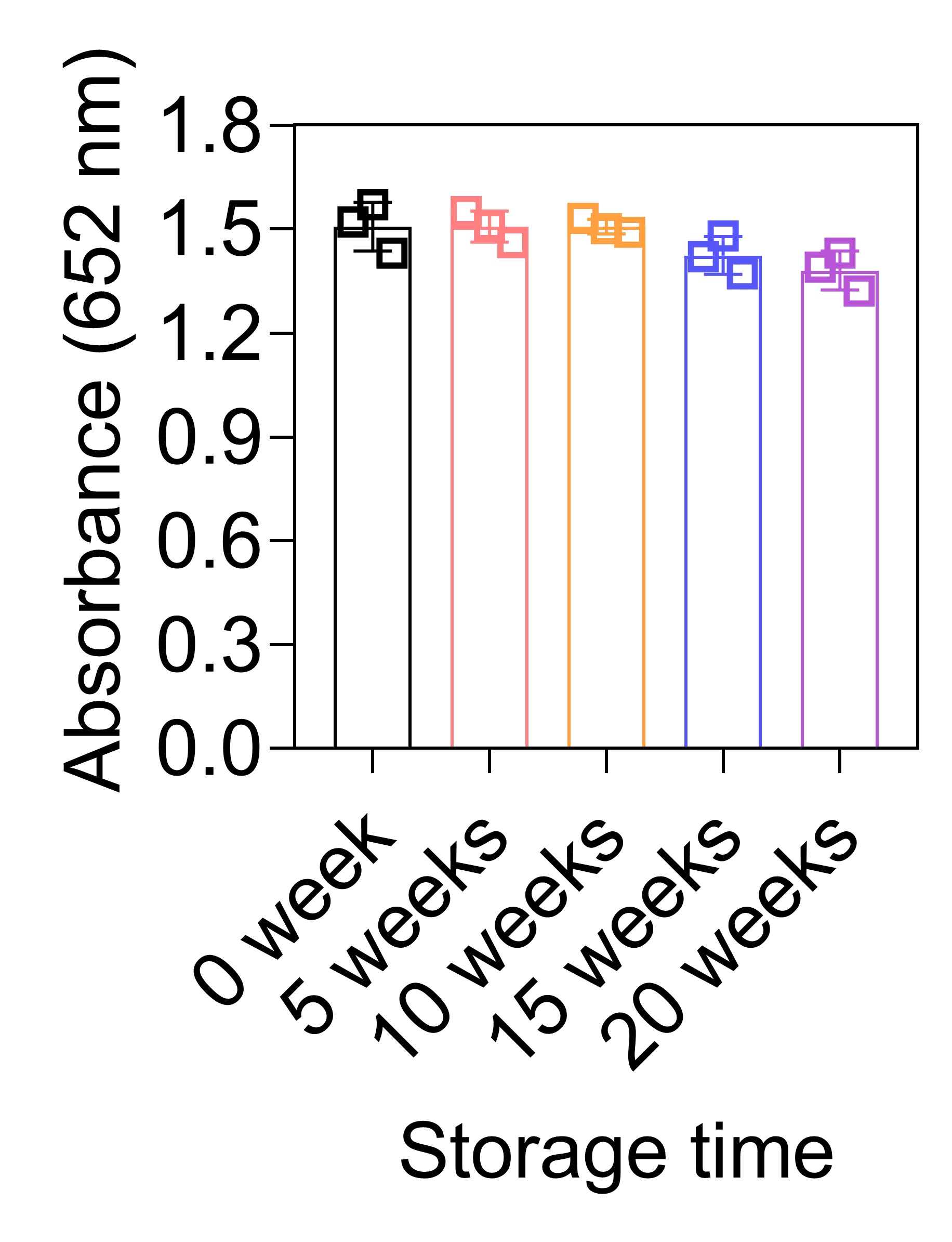


**Figure S8.** The stability test for Ru-C_3_N_4_ under room temperature.


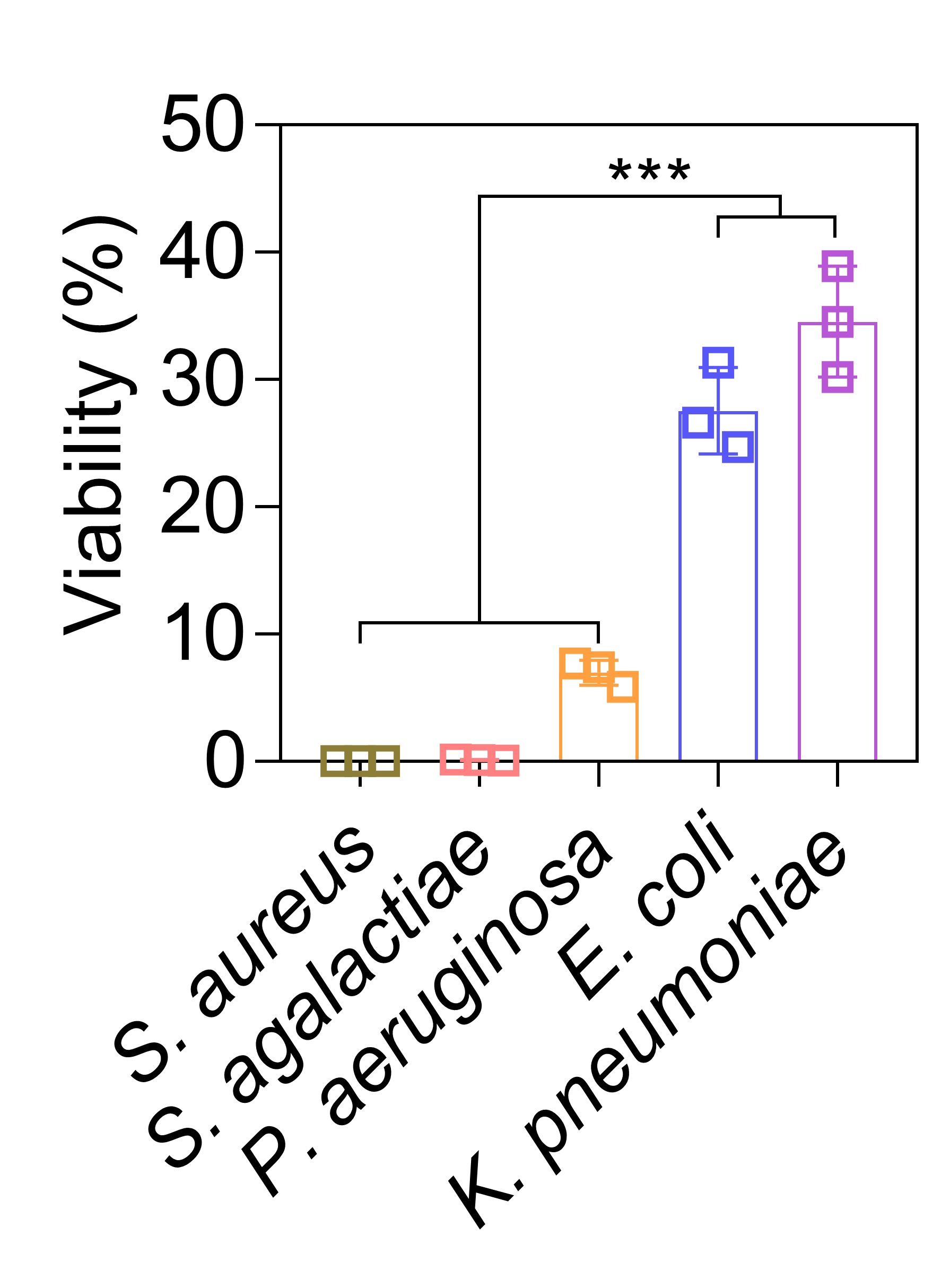


**Figure S9.** Comparison of the antibacterial efficiency of Ru-C_3_N_4_ against Gram-positive and Gram-negative bacteria.


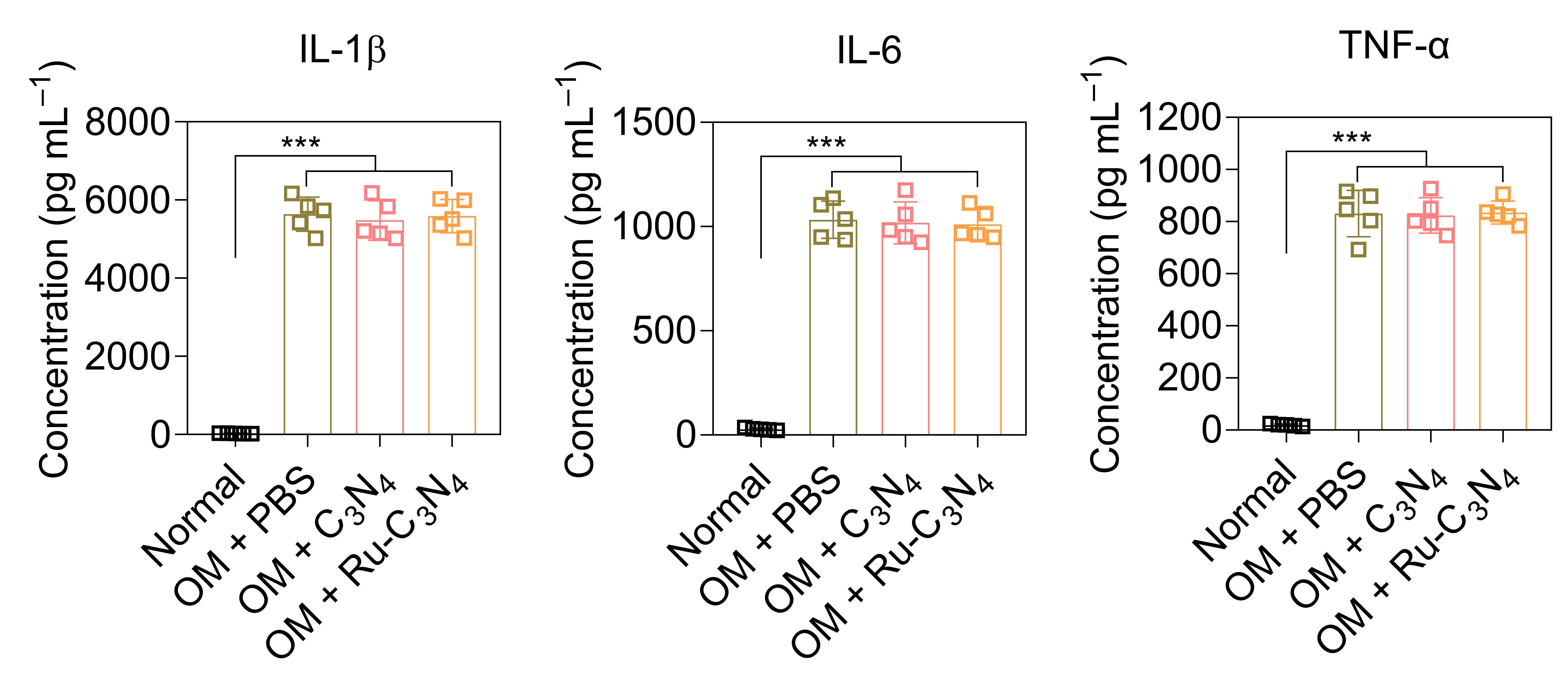


**Figure S10.** Inflammatory cytokines detection in the middle ear lavage fluid of OM model at day 0.


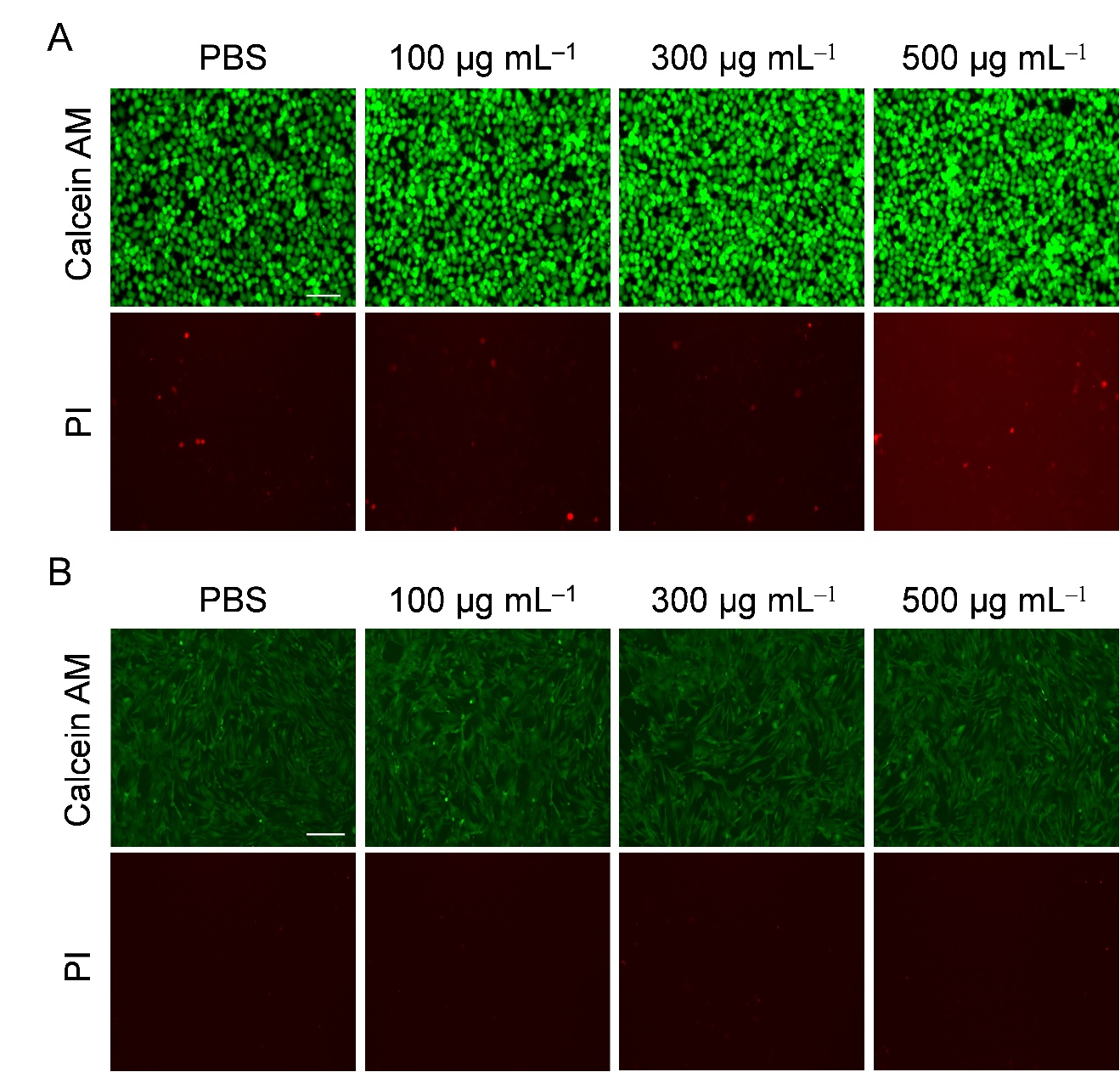


**Figure S11.** The biosafety evaluation of Ru-C_3_N_4_ in vitro. **(A)** Calcein AM/PI staining for HUVEC treated with100-500 μg mL^–1^ Ru-C_3_N_4_. Scale bar = 100 μm. **(B)** Calcein AM/PI staining for HSF treated with100-500 μg mL^–1^ Ru-C_3_N_4_. Scale bar = 300 μm.


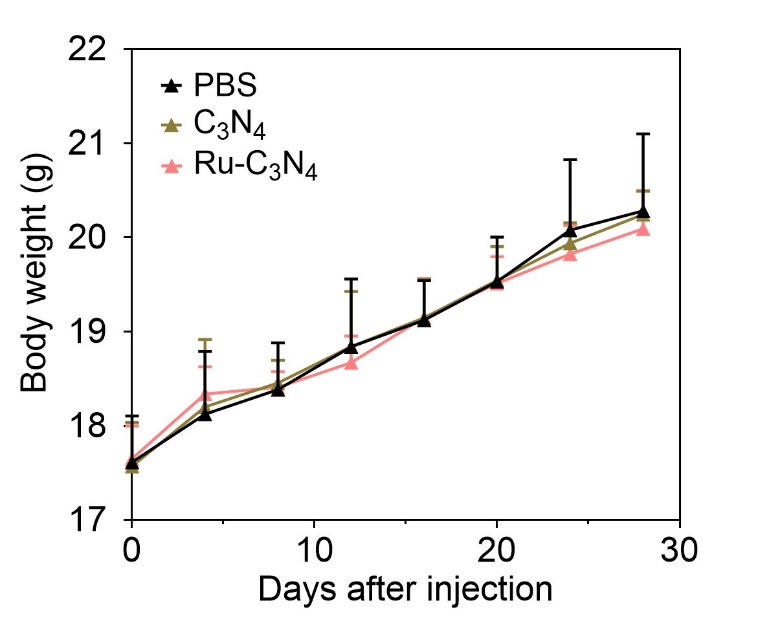


**Figure S12.** The body weight of mice treated with C_3_N_4_ and Ru-C_3_N_4_.


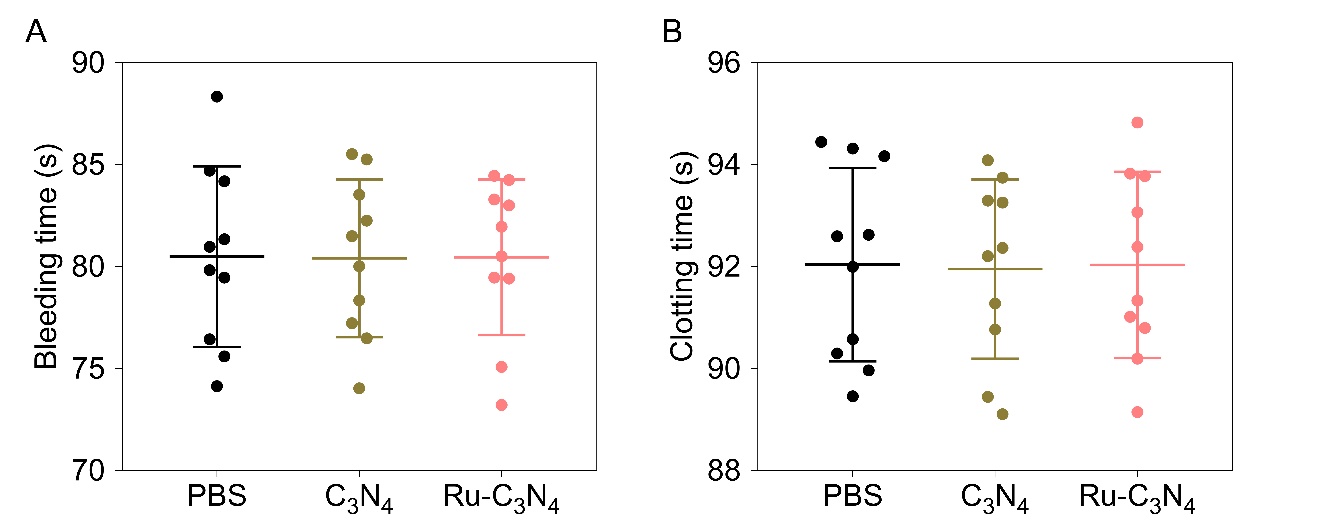


**Figure S13. (A)** The bleeding time and **(B)** clotting time of mice treated with C_3_N_4_ and Ru-C_3_N_4_.


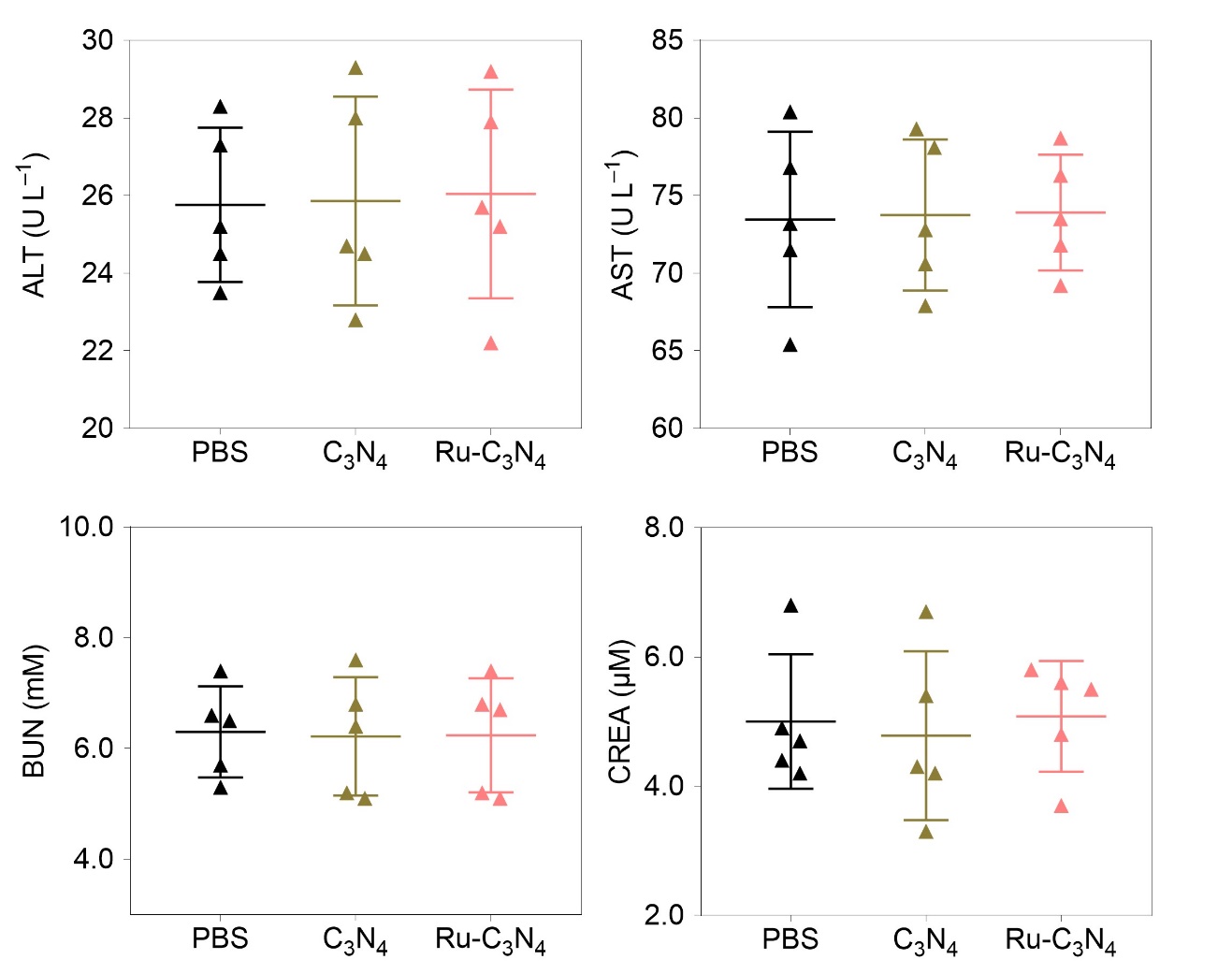


**Figure S14.** In vivo hematological parameters detection. ALT: alanine transaminase, AST: aspartate aminotransferase, BUN: blood urea nitrogen, CREA: creatinine.

## Supplementary Table

**Table S1.** The loading amount of metal atoms on C_3_N_4_ by ICP-OES.

| **Nanomaterials** | **Metal element** | **Weight%** |
| --- | --- | --- |
| Ru-C_3_N_4_ | Ru | 1.12 |
| Fe-C_3_N_4_ | Fe | 1.88 |
| Cu-C_3_N_4_ | Cu | 0.95 |
| Ag-C_3_N_4_ | Ag | 0.66 |

**Table S2.** The kinetics parameters of Ru-C_3_N_4_ and HRP.

| **Materials** | **[E/Ru]**  **(M)** | ***K*_M_**  **(M)** | ***V*_max_**  **(M s^–1^)** | ***k*_cat_**  **(s^–1^)** | ***k*_cat_/*K*_M_**  **(M^–1^s^–1^)** |
| --- | --- | --- | --- | --- | --- |
| Ru-C_3_N_4_ | 4.17 × 10^–9^ | 7.65 × 10^–5^ | 8.42 × 10^–6^ | 2.02 × 10^3^ | 2.64 × 10^7^ |
| HRP | 2.27 × 10^–9^ | 9.42 × 10^–5^ | 8.72 × 10^–6^ | 3.84 × 10^3^ | 4.08 × 10^7^ |
